# Age-related differences in the effect of chronic alcohol on cognition and the brain: a systematic review

**DOI:** 10.1038/s41398-022-02100-y

**Published:** 2022-08-25

**Authors:** Lauren Kuhns, Emese Kroon, Heidi Lesscher, Gabry Mies, Janna Cousijn

**Affiliations:** 1 Neuroscience of Addiction (NofA) Lab, Department of Psychology, University of Amsterdam, Amsterdam, The Netherlands; 2 The Amsterdam Brain and Cognition Center (ABC), University of Amsterdam, Amsterdam, The Netherlands; 3 Department of Animals in Science and Society, Division of Behavioural Neuroscience, Faculty of Veterinary Medicine, Utrecht University, Utrecht, The Netherlands; 4 Department of Psychology, Education & Child Studies, Erasmus University Rotterdam, Rotterdam, The Netherlands

**Keywords:** Addiction, Human behaviour

## Abstract

Adolescence is an important developmental period associated with increased risk for excessive alcohol use, but also high rates of recovery from alcohol use-related problems, suggesting potential resilience to long-term effects compared to adults. The aim of this systematic review is to evaluate the current evidence for a moderating role of age on the impact of chronic alcohol exposure on the brain and cognition. We searched Medline, PsycInfo, and Cochrane Library databases up to February 3, 2021. All human and animal studies that directly tested whether the relationship between chronic alcohol exposure and neurocognitive outcomes differs between adolescents and adults were included. Study characteristics and results of age-related analyses were extracted into reference tables and results were separately narratively synthesized for each cognitive and brain-related outcome. The evidence strength for age-related differences varies across outcomes. Human evidence is largely missing, but animal research provides limited but consistent evidence of heightened adolescent sensitivity to chronic alcohol’s effects on several outcomes, including conditioned aversion, dopaminergic transmission in reward-related regions, neurodegeneration, and neurogenesis. At the same time, there is limited evidence for adolescent resilience to chronic alcohol-induced impairments in the domain of cognitive flexibility, warranting future studies investigating the potential mechanisms underlying adolescent risk and resilience to the effects of alcohol. The available evidence from mostly animal studies indicates adolescents are both more vulnerable and potentially more resilient to chronic alcohol effects on specific brain and cognitive outcomes. More human research directly comparing adolescents and adults is needed despite the methodological constraints. Parallel translational animal models can aid in the causal interpretation of observed effects. To improve their translational value, future animal studies should aim to use voluntary self-administration paradigms and incorporate individual differences and environmental context to better model human drinking behavior.

## Introduction

Alcohol use disorder (AUD) is the most prevalent substance use disorder worldwide [1]. Most AUDs remain untreated [2] and for those seeking treatment, relapse rates are high [3]. Adolescence marks a rapid increase in AUD and an earlier onset of AUD is associated with worse long-term outcomes, including greater problem severity and more relapses [4, 5]. Loss of control over alcohol use is a core aspect of AUD [6] and the developmentally normative difficulty to control motivational urges in tempting and arousing situations is thought to put adolescents at risk for developing addictive behaviors [7]. Moreover, neurotoxic consequences of alcohol use may be more severe for a developing brain [8]. Paradoxically, adolescence is also a period of remarkable behavioral flexibility and neural plasticity [9–11], allowing adolescents to adapt their goals and behavior to changing situations [12] and to recover from brain trauma more easily than adults [10]. In line with this, the transition from adolescence to adulthood is associated with high rates of AUD recovery without formal intervention [13]. While the adolescent brain may be a vulnerability for the development of addiction, it may also be more resilient to long-term effects compared to adults. Increased neural plasticity during this period could help protect adolescents from longer-term alcohol use-related cognitive impairments across multiple domains, from learning and memory to decision-making and cognitive flexibility. Therefore, the goal of this systematic review was to examine the evidence of age-related differences in the effect of alcohol on the brain and cognitive outcomes, evaluating evidence from both human and animal studies.

**Fig. 1:**
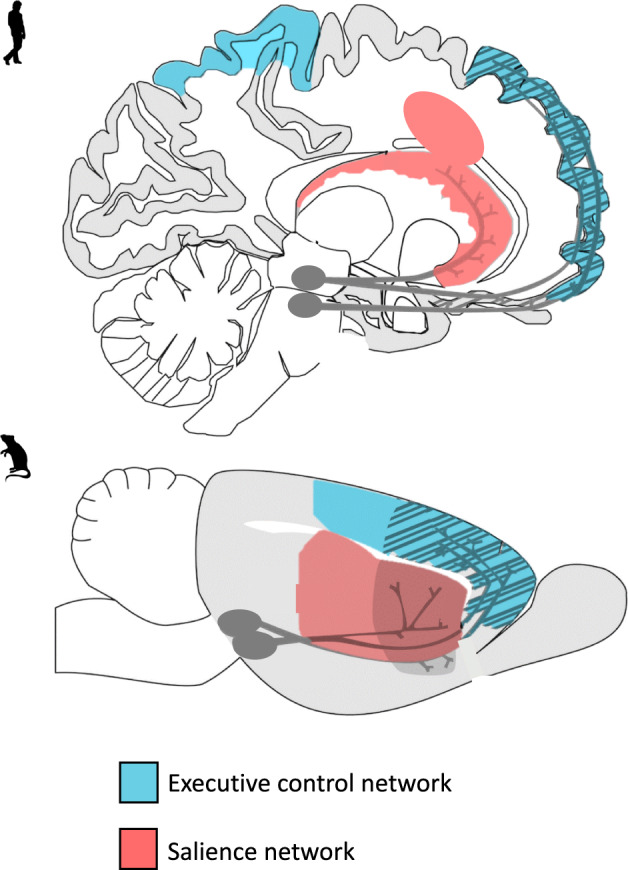
Translational brain models of addiction. A visual representation of the translational model of the executive control and salience networks in humans and rodents. The executive control and salience are key networks believed to play a part in adolescent vulnerability to alcohol-related problems.

In humans, the salience and reinforcement learning network as well as the central executive network are involved in the development and maintenance of AUD [7, 14]. The central executive network encompasses fronto-parietal regions and is the main network involved in cognitive control [15]. The salience network encompasses fronto-limbic regions crucial for emotion regulation, salience attribution, and integration of affective information into decision-making [15, 16], which overlaps with fronto-limbic areas of the reinforcement learning network (Fig. 1). Relatively early maturation of salience and reinforcement learning networks compared to the central executive network is believed to put adolescents at heightened risk for escalation of alcohol use compared to adults [7]. Rodent models are regularly used for AUD research and allow in-depth neurobehavioral analyses of the effects of ethanol exposure during different developmental periods while controlling for experimental conditions such as cumulative ethanol exposure in a way that is not possible using human subjects because exposure is inherently confounded with age. For example, animal models allow for detailed neurobiological investigation of the effects of alcohol exposure in a specific age range on neural activation, protein expression, gene expression, epigenetic changes, and neurotransmission in brain regions that are homologous to those that have been implicated in AUD in humans.

While most of our knowledge on the effects of alcohol on the brain and cognitive outcomes is based on research in adults, several recent reviews have examined the effects of alcohol on the brain and cognition in adolescents and young adults specifically [17–25]. Heavy or binge drinking has been associated with reduced gray and white matter. Also, altered task-related brain activity [20], structural abnormalities [25], and overlapping behavioral impairment in executive functioning have been identified in adolescent and young adult alcohol users [19]. While some of the observed neurocognitive differences between drinkers and non-drinkers may be predisposing factors, they may be further exacerbated by heavy and binge drinking [21, 23]. Furthermore, reviews of longitudinal studies concluded that adolescent alcohol use is associated with neural and cognitive alterations in a dose-dependent manner [17, 22].

Although previous reviews underscore the potential negative consequences of heavy alcohol use on the brain and cognition in adolescence, they do not typically address the question of whether adolescents are differentially vulnerable compared to adults to the effects of alcohol on these outcomes. Explicit comparisons between adolescents and adults are crucial to identify potential risk and resilience factors. In the current review, we aimed to extend previous work by systematically examining this critical question: does the relationship between chronic alcohol use and neurocognitive outcomes differ *between* adolescents and adults? To address this question, we systematically reviewed human and animal studies that included both age groups and used a factorial design that would allow for the comparison of the effects of chronic alcohol use on cognitive and brain-related outcomes across age groups. We specifically highlight outcomes from voluntary self-administration paradigms when available and discuss the translational quality of the animal evidence base. We conclude with a discussion of prominent knowledge gaps, future research directions, and clinical implications.

## Methods

### Study inclusion criteria and search strategy

We followed the PRISMA guidelines for the current systematic review (The PRIMSA Group, 2009). An initial MedLine, Cochrane Library, and PsycInfo search was conducted during September of 2018 with terms related to alcohol, cognition, adolescence/adulthood, and study type (see Appendix for full search strategy and syntax). Two search updates using the same search strategy were conducted on 31 March 2020 and 3 February 2021. For all searches, the identified citations were split into batches and at least two of the following assessors (GM, LK, JC, or CG) conducted a blinded review to determine whether articles met the inclusion criteria. In the first phase of screening, only titles and abstracts were screened and articles that clearly did not meet the inclusion criteria were excluded. In the second phase, the remaining articles received a full-text review and those that did not meet all inclusion criteria were excluded. The first inclusion criterion that was not adhered to was recorded as the reason for excluding. If there was a discrepancy between authors after initial and full-text screening process, the reviewing authors discussed the article and a consensus was reached.

**Fig. 2:**
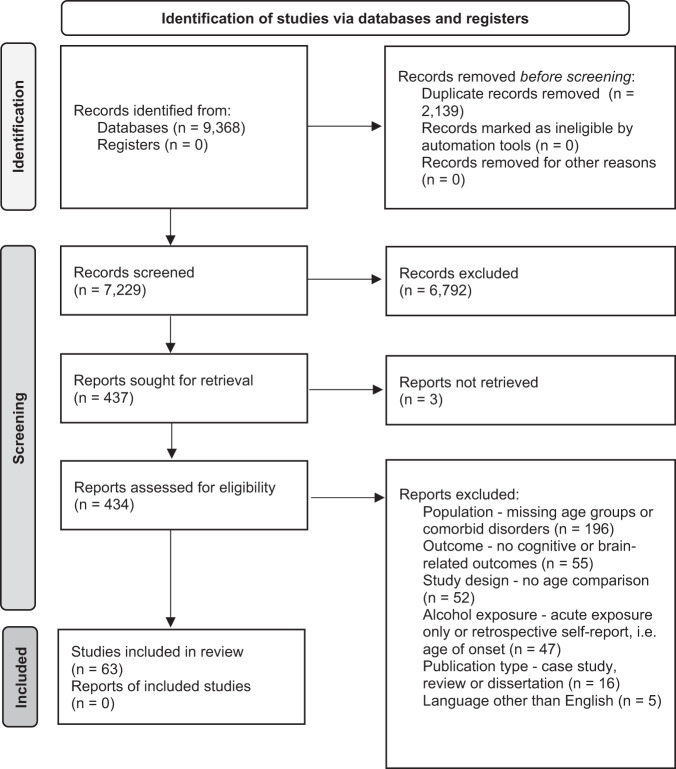
PRIMSA flow diagram detailing the screening process.

The inclusion criteria were: (1) Human samples including both adolescents younger than 18 and adults older than 18 and animal samples including adolescent (Post Natal Day (PND) 25–42 for rodents) and adult [8] animals (greater than PND 65 for rodents); (2) Exploration of alcohol as the independent variable and cognitive, reward-related, or brain outcomes as the dependent variables; (3) Alcohol and cognitive outcomes must meet our operationalization defined below; (4) Study design comparing adults and adolescents on outcome measures; (5) Administering or measuring alcohol use *during* adolescence or adulthood, not retrospectively (e.g., no age of onset work in humans using retrospective self-reports of alcohol consumption); (6) Primary quantitative data collection (no case studies, or review papers); (7) Solely looking at alcohol-related factors as the independent variables (e.g., cannot explore alcohol-related factors in individuals with psychosis); (8) Written in English; (9) Published in a peer-reviewed journal before February 3, 2021 (see Fig. 2 for a detailed screening process).

The definitions for adolescence are variable, hampering the direct comparison of human and rodent research. In rodents, the end of early-mid adolescence is considered to be approximately PND 42 when rats reach sexual puberty. By contrast, the boundaries for the onset of early adolescence are less clear. Based on the notion that most age-typical physiological changes that are characteristic of adolescence emerge from PND 28 [26], the conservative boundary for adolescence has been set at PND 28 (e.g., seminal review on adolescence [27]). The preceding week (PND 21-PND 28) has been described as the juvenile period (e.g., [28, 29]) but these same reports consider PND 21-PND 23 as the lower boundary for early adolescence [28, 29], further emphasizing that the boundary of PND28 may be too conservative. Indeed, multiple studies (e.g., [30, 31]), have chosen to take PND25 as the boundary for early adolescence. Hence, we have decided to also follow this less conservative approach and include all studies where alcohol was administered between PND 25 and PND 42.

The exact boundaries of human adolescence are similarly nebulous. From a neurodevelopmental perspective, adolescence is now often thought of as continuing until approximately age 25 because of the continuing maturation of the brain [32]. However, the delineation of adolescence and adulthood is also dependent on societal norms, and is commonly defined as the transitional period between puberty and legal adulthood and independence which typically begins around age eighteen. In light of this, we chose a relatively liberal inclusion criteria for the human studies; studies needed to include at least some adolescents below eighteen, the age at which drinking typically begins, as well as ‘adult’ participants over the age of eighteen. We are careful to interpret the results of human studies within the neurodevelopmental framework of adolescence, such that 18–25-year-olds are considered late adolescents to young adults who are still undergoing cognitive and brain maturation.

Notably, we excluded studies that assessed alcohol exposure retrospectively (primarily early onset alcohol studies) because age of onset variables are often inaccurate, with reported age of alcohol onset increasing with both historical age [33] and current alcohol use patterns [34]. In addition, we excluded work that has not undergone peer-review to ensure high-quality papers.

In humans, we defined cognition as any construct that typically falls within the umbrella of neuropsychological testing, as well as brain-based studies. We also included more distal constructs of cognition, like craving and impulsivity, because they play a prominent role in addictive behaviors [35, 36]. In rodents, we defined cognition as attention, learning, and memory in line with a seminal review paper [37]. Given the importance of social cognition in patterns of alcohol use particularly in adolescence [38] and its proposed role in adolescent risk and resilience to addiction [39], we included social behavior as an outcome. Furthermore, because many rodent studies assessed anxiety-related behaviors and the high degree of comorbidity between anxiety disorders and alcohol addiction [40], we also included anxiety as a secondary outcome. On the other hand, locomotor activity was excluded as an outcome because even though behavioral sensitization is considered to reflect neurobiological changes that may underlie certain aspects of addictive behavior [36], the translational relevance for addictive behavior and human addiction in particular remains unclear [41, 42]. Across both rodents and humans, general alcohol metabolization and ethanol withdrawal studies were not included except if they included brain-related outcomes. The relevant reported findings (i.e., the results of an analysis of comparing age groups on the effect of alcohol on an included outcome) were extracted by a one reviewer and then confirmed by at least one other reviewer. In addition, the characteristics of the sample, details of alcohol exposure, and study design were extracted by a single reviewer and then confirmed by at least one other reviewer. No automation tools were used for extraction. Within the included studies, peripheral findings that did not relate to cognition were excluded from review and not extracted. The protocol for this systematic review was not registered and no review protocol can be accessed.

## Results

### Study search

Our searches identified 7229 studies once duplicates were removed. A total of 6791 studies were excluded after initial review of abstracts. Then, 434 studies received a full-text review and 371 were excluded for failing to meet all inclusion criteria. See Fig. 2 for a flow diagram of the full screening process. At the end of the inclusion process, 59 rodent studies and 4 human studies were included. The characteristics and findings of the final studies are detailed in Table 1 (rodents) and Table 2 (humans). Due to the heterogeneity of outcomes, meta-regression was not suitable for synthesizing results. Results are narratively synthesized and grouped based on forced or voluntary ethanol exposure and by outcome within the tables and by outcome only in text. Two authors independently rated the quality of evidence for human studies (Table 2) based on criteria used in a similar systematic review [43]: (1) strong level of causality: longitudinal design comparing adolescent and adults while adjusting for relevant covariates; (2) moderate level of causality: longitudinal design comparing adolescents and adults without adjusting for relevant covariates or cross-sectional designs with matched groups that considered relevant covariates; (3) weak level of causality: cross-sectional design without matched adolescent and adult groups and/or did not adjust for relevant covariates. A methodological quality assessment was not conducted for the animal studies due to a lack of empirically validated risk of bias tools and lack of standardized reporting requirements in the animal literature.

**Table 1:** Characteristics and findings of animal studies on age-related differences on the effect of alcohol on cognition and the brain.

	Sample	Characteristics of EtOH exposure	Outcomes	Design	Result
*Voluntary exposure*					
Schramm-Sapyta et al., 2010	Male Sprague-Dawley rats; *N* = 34–38 Adolescents PND 28, *N* = 34–38 Adults PND 65	16 h/day, water in both bottles on day 1–3, 10% (v/v) EtOH in both bottles day 4–6, 8% (v/v) EtOH in one bottle day 7–16, 16 days; 2d abstinence followed by choice between 8% (v/v) EtOH and water. Note: this procedure started at PND 35 & 72 and followed an i.p. injection (0.0, 0.5, 1.0 or 3.5 g/kg EtOH (20% v/v) or saline) during conditioned taste aversion.	CTA	Age X Treatment	Adolescents ↓ aversion than adults at low but not high dose
			EtOH consumption and preference	Age, Age X CTA score	Adolescents ↑ than adults during EtOH-only phase and after 2 days of deprivation; Adolescents with lower CTA ↑ EtOH consumption after deprivation
Labots et al., 2018	Male Lister Hooded rats; *N* = 84 Adolescents PND 42, *N* = 84 Adults PND 77; Note: Each age group split into low, medium, and high drinkers based on voluntary consumption	7 h/day, 3d/week, 20% (v/v) EtOH, during month 1; 24 h/day, 3d/week, 20% (v/v) EtOH, during month 2; abstinence not specified	EtOH consumption and preference over water	Age X Group X Month	EtOH consumption and preference escalation in month 2, especially in adults↑
			Conditioned suppression of EtOH-seeking	Age X Conditioning X Group X Tone X Interval	Conditioned suppression of EtOH seeking in low drinking adults, but not in medium and high drinking adults; Conditioned suppression in medium and high drinking adolescents, but not in low drinking adolescents
Pickens et al., 2019	Male Long Evans rats; *N* = 24 Adolescents PND 26, *N* = 36 Adults PND 68	24 h/day, 3x/week, 24–48 h interval, 20% (v/v) EtOH solution and water in separate bottles (last two days water only), 6 weeks; 17d abstinence for adolescents and 10d abstinence for adults; Note: Adolescents free-fed during EtOH access period, adults EtOH access period crossed with food restriction	Sign tracking and omission contingency learning	Treatment X Lever X Training Day	Sign-tracking: no effect of treatment in either age; Contingency learning: EtOH-adolescents ↑ omission contingency learning vs. EtOH-adults
			EtOH consumption	Access group X Week	Adult-food restricted↑ vs. adolescents; over time EtOH consumption escalated only in the adult groups, not in the adolescent group
Schindler et al., 2014	Male Sprague-Dawley rats; *N* = 6–7/group Adolescents PND 30, *N* = 8–10/per group Adults PND 80	24 h/day, 10% EtOH gel or water, 20 days; 20d abstinence	EtOH consumption	Age X Time	Overall adolescents ↔ adults; initially adolescents ↑
			Decision-making; risk-taking behavior	Age, Treatment	Risky decision-making EtOH-exposed adolescents ↑ vs. age-matched controls; Risky decision-making EtOH-exposed adults ↔ age-matched controls
Agoglia et al., 2015	Male C57BL/6J mice; *N* = 20 Adolescents PND 28, *N* = 20 Adults PND 70	4 h/day, 20% (v/v) EtOH or water, 2 weeks; no abstinence	EtOH consumption	Age X Treatment	Adolescents ↔ adults
			Neurotransmission; CaMKIIα & GluA1 levels in amygdala & NAc	Age, Treatment	Adolescent amygdala: CaMKIIαT286 ↓ but CaMKIIα, GluA1 & GluA1Ser831 ↔; Adult amygdala; GluA1Ser831 ↑ but CaMKIIαT286, CaMKIIα & GluA1 ↔; NAc: no efffects
	Male C57BL/6J mice; *N* = 12 Adolescents PND 28, *N* = 12 Adults PND 70	4 h/every other day, 20% (v/v) EtOH or 0.5% sucrose, 2 weeks; 4d abstinence; Note: tianeptine (antidepressant acting on CaMKIIα) pretreatment after week 1 (0, 3, 10, or 17 mg/kg)	EtOH consumption	Age X Day; Age x Dose	EtOH consumption adolescents ↑; tianeptine pretreatment EtOH consumption adolescents ↑ adults↓
			Anxiety; OFT	Age X Dose	No effects on anxiety behavior
Lee et al., 2016	Male C57BL/6J mice; *N* = 24 Adolescents PND 28, *N* = 24 Adults PND 56	2 h/day, 5%, 10%, 20% and 40% (v/v) EtOH or water, 2 weeks; no abstinence	EtOH consumption	Age x Day	Adolescents ↑
			Neurotransmission; levels of mGlu1, mGlu5, GluN2A, GluN2B, CAMKII, CaMKIIαT286, PKCε, pPKCεS729 levels in the NAc core and NAc shell	Age X Treatment	NAc shell: baseline mGlu1 & mGlu5 adolescents ↑, EtOH in adults mGlu1, mGlu5, GluN2B ↑; NAc core: EtOH in adults GluN2B, PKCε & CAMKII ↑
			Anxiety; NOT, MBT	Age X Treatment	EtOH activity novel object test ↓, no age x treatment interaction; EtOH marble burying in adults ↑ & adolescents ↓
Wille-Bille et al., 2017	Male & female Wistar rats; *N* = 20 Adolesents PND 25, *N* = 20 Adults PND 80	18 h/day, 3d/week, 5% (w/v) EtOH (mixed with 1% sucrose in week 1, with 0.5% sucrose in week 2, and with plain water in weeks 3–6) or sucrose/water (1% sucrose in week 1, 0.5% in week 2, plain water in weeks 3–6), 6 weeks; 48 h abstinence; Note: 3d of additional abstinence for L-D box test	EtOH consumption and preference over vehicle	Age X Day X Sex	Adults ↑, but age difference gradually faded away at the end
			Anxiety; L-D box	Age X Treatment X Sex	No age effects reported
			Neural plasticity; ΔFosB immunoreactivity in mesocorticolimbic pathway regions	Age X Treatment X Sex	Raw number: EtOH-exposed adolescents ↑ in prelimbic prefrontal area, dorsomedial striatum, NAc core and shell, central amygdala nucleus capsular and BLA; No age effects in dorsolateral striatum & lateral orbital cortex; Percentage change: Adolescents ↑ in all regions except for central amygdala nucleus capsular
Agrawal et al., 2014	Offspring of bred FVB/NJ mice: *N* = 30 Adolescents PND 30, *N* = 39 Adults PND 70; offspring of bred C57BL/6J mice: *N* = 30 Adolescents PND 30, *N* = 64 Adults PND 70	4 h/day, 20% (v/v) EtOH solution (water access for remaining 20 h), 4 days; no abstinence; Note: i.p. injection of minocycline (50 mg/kg) or saline daily during DID procedure	EtOH consumption & neuroimmune; full transcriptome gene expression	Consumption per sex: Age X Days; Transcriptome in male: age and treatment effects; Minocycline: Age X Sex X Treatment	Male EtOH-adolescents ↑ consumption vs. male adults; no age differences in females (not included in transcriptome analyses); Over-representation of changes related to microglia action in EtOH-adults vs. adolescents: toll-like Receptor Signaling, MAPK Signaling, Jak-STAT Signaling, T-Cell Signaling, and Chemokine Signaling; Minocycline identified as therapeutic target: Minocycline-EtOH-adults only ↓ voluntary drinking; no sex differences
Hargreaves et al., 2009	Male albino Wistar rats; *N* = 12 Adolescents PND 27, *N* = 12 Adults PND 55; Note: highest drinking rats selected for analysis	8 h/day for day 1–10, 8 h/every other day for next 22 days, beer (increase from 0.44 to 3.44% (v/v) EtOH in first 4 days and was 4.44% on later days), 4 weeks; 2w abstinence	Protein expression in the HC	Age X Treatment	EtOH-exposed adolescents: protein expression ↓ related to glutamate metabolism, signaling/cell cycle, glycolysis, cellular degradation/neurodegeneration, and cytoskeletal processes; EtOH-exposed adults: metabolic (Krebs cycle) protein expression ↑
*Forced exposure*					
Holstein et al., 2011	Male C57BL/6J mice; *N* = 30–36 Adolescents PND 29, *N* = 30 Adults PND 71	i.p. injection of 0, 3 or 4 g/kg EtOH on day 10, 12, 14 & 16, 33 days (4 trials total); Note: extinction testing after 6d, 11d, and 16d abstinence	CTA	Age X Treatment Dose X Conditioning trial	Adolescents (4 g/kg) ↑ dose than adults (3 g/kg) to develop CTA. Adults ↓ extinction of CTA to 4 g/kg
Moore et al., 2013	Eight inbred mouse strains (C57BL/6J, DBA/2J, 129S1/SvImJ, A/J, BALB/cByJ, BTBR T + tf /J, C3H/HeJ and FVB/NJ); *N* = 8–12/strain Adolescents PND 30, *N* = 8–12/strain Adults PND 75	i.p. injection of 0, 1.5, 2.25 or 3 g/kg EtOH or saline on day 2, 5, 8, 11 & 13, 14 days (5 trials total); Note: outcome measured just before every injection	CTA	Age X Treatment Dose X Genotype X Day	In 6 of 8 strains, adolescents ↑ dose than adults to develop CTA.
Pautassi et al., 2011	Male and female Sprague-Dawley rats; *N* = 109 Adolescents PND 30, *N* = 118 Adults PND 68	i.g. injection of 3 g/kg (adults) or 3.25 g/kg (adolescents) EtOH or saline on 1, 2 or 3 consecutive days (1–3 trials total); 1d abstinence	CTA, SOPC	Age X Treatment Dose X Sex X # of trials	Adolescents ↔ Adults; no sex differences
Carrara-Nascimento et al., 2014	Male Swiss mice; *N* = 27 Adolescents PND 33–35, *N* = 24 Adults PND 65–67	Pre-treatment: i.p. injection of 2 g/kg 20% (v/v) EtOH or saline daily, 15 days; 5d abstinence; CPP treatment: i.p. injection of 2 g/kg (20% v/v) EtOH or saline alternated daily, 8 days (4 injections total)	CPP	Age X Pre-Treatment X Compartment	EtOH-Adolescents ↑ CPP vs. adults; EtOH-Adults ↓ CPP vs. controls
Leichtweis et al., 2020	Male and female Wistar rats; *N* = 40 Adolescents PND 28, *N* = 40 Adults PND 70; Note: this is a subset of non-maternally separated control animals from study of maternal separation	i.p. injection of 0.5 g/kg or 1 g/kg EtOH every other day, 8 days (4 injections total); 2d abstinence	CPP	Sex X Test phase: pre- or post-conditioning	No CPP in adults or adolescents for either dosage
Pascual et al., 2012	Male Wistar rats; *N* = 32 Adolescents PND 30, *N* = 32 Adults PND 90	i.p. injection of 3 g/kg 25% (v/v) EtOH or saline, 2d on - 2d off, 16 days (8 injections total total); 1d or 14d abstinence	Epigenetics; HAT & DHAC expression, histone acetylation cFos, FosB, Cdk5, BDNF in the PFC	Age X Treatment	1 day but not 14 day abstinence EtOH-Adolescents HAT ↑, no effect HDAC; 1 day abstinence EtOH-Adolescents ↑ H3 acetylation cFos, H4 acetylation FosB, Cdk5, BDNF, H3 dimethylation FosB but EtOH-adults ↑ H3 dimethylation BDNF; 14 day abstinence adolescent EtOH ↑ mRNA FosB, no effects mRNA BDNF, cFos, Cdk5
	Male Wistar rats; *N* = 96 Adolescents PND 30, *N* = 96 Adults PND 90		CPA	Age X Treatment X Conditioning dose	No EtOH or age effects on CPA
Pautassi et al., 2017	Male Swiss mice; Experiment 3: *N* = 38 Adolescents PND 43, *N* = 41 Adults PND 85	i.p. injection of 2 g/kg 16.8% (v/v) EtOH or saline alternated daily, 8 days (4 injections total); 1d abstinence	BDNF levels in the PFC	Age X Housing conditions X Treatment	EtOH-adolescents (standard care) ↓ in PFC vs. adults (standard care)
	Male Swiss mice; Experiment 1: *N* = 37 Adolescents PND 43; Experiment 2: *N* = 39 Adults PND 85	i.p. injection of 2 g/kg 16.8% (v/v) EtOH or saline alternated daily, 8 days (4 injections total); 1d, 3d, 5d or 6d abstinence	CPP	Age, Housing conditions X treatment	EtOH-adolescents CPP in environmentally enriched housing conditions, while Adults show CPP in both enriched and standard conditions
Bergstrom et al., 2006	Male and female Long-Evans hooded rats; *N* = 40 Adolescents PND 28, *N* = 40 Adults PND 80	1 h/day (other 22.5 h/day water deprivation), 10% (v/v) EtOH solution, 18 days; 30d abstinence	EtOH consumption	Age X Treatment X Gender	Adolescents ↑ adults, female adolescents ↑ male adolescents
			Fear conditioning; tone	Age X Treatment X Gender X Minute	Adolescents (m/f) only ↓ compared to controls
Broadwater and Spear, 2013	Male Sprague-Dawley rats; Experiment 1: *N* = 36–48 Adolescents PND 28, *N* = 36–48 Adults PND 70; Experiment 2: *N* = 48 Adolescents PND 35	i.g. injection of 4 g/kg EtOH or water every other day, 20 days (11 injections total); 22d abstinence	Fear conditioning; tone and context	Age X Treatment X Conditioning stimulus	No effect in tone conditioning, retention, or extinction; Mid-Adolescents ↓ context fear retention vs. adults; Adults ↑ fear extinction vs. Mid-Adolescents; Late Adolescents ↔ Adults
Broadwater and Spear, 2014	Male Sprague-Dawley rats; *N* = 64–80 Adolescents PND 28, *N* = 64–80 Adults PND 70	i.g. injection of 4 g/kg 25% (v/v) EtOH or water every other day, 20 days (11 injections total); 22d abstinence followed by i.p. injection of 1 g/kg (20% v/v) EtOH or saline 10 min before testing	Fear conditioning; tone and context	Age X Treatment X Conditioning stimulus X Acute challenge	Adolescents ↔ Adults acute EtOH ↓ tone and context retention; no effect of chronic exposure history; EtOH-Adolescents ↓ freezing than adults
Broadwater and Spear, 2014	Male Sprague-Dawley rats; *N* = 64–96 Adolescents PND 28, *N* = 64–96 Adults PND 70	i.g. injection of 4 g/kg EtOH or water every other day, 20 days (11 injections total); 22d abstinence	Fear conditioning; context and tone+context	Age X Treatment X Conditioning stimulus X Acute challenge	Context only: EtOH-Adolescent exposed ↓ than controls & adults, EtOH-Adults ↔ controls; Context + tone: EtOH-adolescents ↑ context fear
Lacaille et al., 2015	Male C57Bl/6J mice; *N* = ? Adolescents PND 30, *N* = ? Adults PND 95; Note: total *N* = 332, groups not specified	i.p. injections of 2.5 g/kg, 2.5 g/kg and 2 g/kg 15% (w/v) EtOH at 2 h intervals every 5 days, 15 days (9 injections total); 8 h abstinence	Neurogenesis & gene expression in FC, striatum, HC, and cerebellum	Age X Treatment	Repair and protection of oxidative DNA damage: EtOH-adults ↔ adolescents ↓ atr, EtOH-adolescents only ↓ gpx7 and nudt15; Proapoptotic genes: EtOH-adolescents only ↑ casp3; Antioxidant genes: EtOH-adults ↔ EtOH-adolescents ↑ mtl and txnip, but EtOH-adults only ↑ gp3 and srxn; Neurogenesis in dentate gyrus: EtOH-adolescents only ↓ BrdU positive cells
		i.p. injections of 2.5 g/kg, 2.5 g/kg and 2 g/kg 15% (w/v) EtOH at 2 h intervals every 5 days, 15 days (9 injections total); 4d abstinence	Short-term memory; NOR	Age X Treatment	EtOH-adolescents only ↓
		i.p. injections of 2.5 g/kg, 2.5 g/kg and 2 g/kg 15% (w/v) EtOH at 2 h intervals every 5 days, 15 days (9 injections total); 3d abstinence	Long-term memory; passive avoidance	Age X Treatment	EtOH-adults ↔ EtOH-adolescents ↓ avoidance behavior
		i.p. injections of 2.5 g/kg, 2.5 g/kg and 2 g/kg 15% (w/v) EtOH at 2 h intervals every 5 days, 15 days (9 injections total); 24 h abstinence	Working memory; Y-maze	Age X Treatment	No age or treatment effects on Y-maze performance
Acheson et al., 2001	Male Long-Evans hooded rats; *N* = 15 Adolesents PND 30, *N* = 15 Adults PND 65	i.p. injection of 0.5 g/kg, 2.5 g/kg EtOH or saline 30 min before training session daily, 4 days, No abstinence	Spatial learning; MWM	Age X Dosage X Day	2.5 g/kg impairs spatial acquisition and retrieval Adolescents ↔ Adults; 0.5 g/kg enhanced acqusition Adolescents ↔ Adults, no effect on retrieval
		i.p. injection of 0.5 g/kg, 2.5 g/kg EtOH or saline 30 min before training session daily, 4 days; 4d abstinence	Non-spatial learning; MWM	Age X Dosage X Day	No effect after controlling for baseline performance in spatial task
Markwiese et al., 1998	Sprague-Dawley rats; *N* = 20 Adolescents PND 30, *N* = 20 Adults PND 65	i.p. injection of 1 g/kg, 2 g/kg EtOH or saline 30 min before training session daily, until memory acquisition; no abstinence	Spatial learning; MWM	Age X Dosage	EtOH-adolescent ↓ control at both dosages; EtOH-adults ↔ control
	Sprague-Dawley rats; *N* = 20 Adolescents PND 30, *N* = 21 Adults PND 65		Non-spatial learning; MWM	Age X Dosage	No effect in either group
Rajendran and Spear, 2004	Male Sprague-Dawley rats; *N* = ? Adolescent PND 26–27, *N* = ? Adults PND 68–70	i.p. injection of 0.5 g/kg, 1.5 g/kg EtOH or saline 30 min before training session daily, 6 days; 1d abstinence	Spatial and non-spatial learning; SBM	Age X Condition (spatial or non-spatial) X dose	No reported age X dose interaction; EtOH-adolescents not impaired in spatial or non-spatial learning; 1.5 g/kg dose adults ↓ spatial learning
Sircar and Sircar, 2005	Sprague-Dawley rats; *N* = 24 Adolescents PND 30, *N* = 24 Adults PND 60	i.p. injection of 2 g/kg EtOH or saline 30 min before training session daily, 5 days; 30 min, 4d, 7d or 25d abstinence	Spatial learning; MWM	Age, Treatment X Day	Adolescents ↔ Adults ↓ learning and memory; Adults only recover after abstinence
Matthews et al., 2019	Male Sprague-Dawley rats, *N* = 30 Adolescents PND 30, *N* = 26 Young adults PND 72, *N* = 30 Aged adults PND 18 months	i.p. injection of 1 g/kg, 2 g/kg 20% (v/v) EtOH or saline every other day, 20 days (10 injections total); 7–8w abstinence	Spatial learning; MWM	Age X Treatment X Training day	No effect of EtOH exposure in either age
		i.p. injection of 1 g/kg, 2 g/kg 20% (v/v) EtOH or saline every other day, 20 day (10 injections total); 6w abstinence	Non-spatial learning; MWM	Age X Treatment X Training day	No effect of EtOH exposure in either age
		i.p. injection of 1 g/kg, 2 g/kg 20% (v/v) EtOH or saline every other day, 20 day (10 injections total); 24 h abstinence	Anxiety; EPM	Age X Treatment	No effect of EtOH exposure on anxiety-like behavior
Swartzwelder et al., 2014	Male Long-Evans hooded rats; *N* = 24 Adolescents PND 30, *N* = 24 Adults PND 70	i.p. injection of 4 g/kg 16.9% (v/v) EtOH or saline daily, 5 days; 2d abstinence followed by i.p. injection of 2 g/kg 12.7% (v/v) EtOH or saline 30 min before each MWM session for 4 days	Spatial learning; MWM	Age, Pre-Treatment X Acute Challenge X Test Day	Spatial learning: no effect of pre-exposure; Thigmotaxis: EtOH-adult ↑ control, while EtOH-adolescent ↔ control; swim speed: EtOH pre-exposed adults ↑
Galaj et al., 2020	Male Sprague-Dawley rats; *N* = 32 Adolescents PND 28, *N* = 32 Adults PND 70	i.g. injection of 4 g/kg 25% (v/v) EtOH or water, 3 day on - 2 days off, 20 days (12 injections total); 2d or 21d abstinence	Reward-related learning; Conditioned reward and approach	Treatment X Age X Abstinence Period X Session	EtOH-adults ↔ EtOH-adolescents ↓ conditioned reward responding vs. controls; No effects on conditioned approach
Fernandez et al., 2016	Male Sprague-Dawley rats; *N* = 35 Adolescent PND 35, *N* = 40 Adult PND 72–75	24 h/day, 6% (v/v) EtOH solution for 4 days (no water), increased by 3% (v/v) every 5 days until reaching 12% (v/v), then increased to 20% and maintained for 28 weeks; T1 group sacrificed while intoxicated; T2 group sacrificed after 2d abstinence; T3 group gradually weened from EtOH for 15 days followed by 3w abstinence	BDNF levels in PFC and HC	Age X Treatment X Time of tissue collection	PFC: EtOH groups ↓; EtOH-Adolescents ↓ vs. controls at intoxication and protracted abstinence time points; EtOH Adults ↓ vs. controls at intoxication, withdrawal, and protracted abstinence; higher blood EtOH concentration ↓ BDNF both ages; HC: no effect of time point or age
			β-NGF in PFC and HC		PFC: EtOH ↓ vs. controls; no age effects; HC: no effect of time, treatment, or age
	Male Sprague-Dawley rats; *N* = 16 Adolescent PND 35, *N* = 16 Adult PND 72–75	24 h/day, 6% (v/v) EtOH solution for 4 days (no water), increased by 3% (v/v) every 5 days until reaching 12% (v/v), then increased to 20% and maintained for 28 weeks; gradually weened from EtOH for 15 days followed by 3w abstinence	Spatial discrimination learning	Age X Treatment	No age or treatment effect after correcting for overall activity
			Non-spatial discrimination and reversal learning (cognitive flexibility)	Age X Treatment	EtOH-adolescents ↓ vs. controls simple discrimination; EtOH ↓ complex discrimination and reversal learning in both ages; no correlation with blood EtOH concentration
			EtOH consumption	Age X Treatment	Adolescents ↑ vs. adults
Fernandez et al., 2017	Male Sprague-Dawley rats; *N* = 69 Early Adolescents PND 28, *N* = 64 Mid-Adolescents PND 35, *N* = 65 Adults PND 65–78	i.g. injection of 5 g/kg 25% (v/v) EtOH or water, 2d on - 2d off, 25 days (13 injections total); T1 sacrificied 1 h after last injection; T2 sacrificed after 2d abstinence; T3 sacrificed after 3w abstinence, T4 sacrificed after 3w abstinence followed by behavioral testing	BDNF in PFC and HC	Age X Treatment X Time of tissue collection	PFC: During intoxication, EtOH exposure ↓ in all ages; During acute abstinence, EtOH-adults ↑, EtOH-early adolescents ↓ vs. controls; No effects in prolonged abstinence; HC: No effects at any time point
	Male Sprague-Dawley rats; *N* = 20 Early Adolescents PND 28, *N* = 20 Mid-Adolescents PND 35, *N* = 19 Adults PND 65–78	i.g. injection of 5 g/kg 25% (v/v) EtOH or water, 2d on - 2d off, 25 days (13 injections total); 3w abstinence	Spatial discrimination learning	Age X Treatment	No age or treatment effect after correcting for overall activity
			Non-spatial discrimination learning and reversal learning (cognitive flexibility)	Age X Treatment	EtOH-adults ↓ vs. controls simple and complex discrimination learning and behavioral flexibility; in simple discrimination task, both ages ↓ flexibility; Blood EtOH concentration negatively correlated with behavioral flexiblity in both ages
Risher et al., 2013	Male Sprague-Dawley rats, *N* = 18 Adolescents PND 30, *N* = 16 Late Adolescents PND 50, *N* = 16 Adults PND 70; Note: Late adolescents and adults were combined in analysis into single adult group	i.g. injection of 5 g/kg 35% (v/v) EtOH, 2d on - 2d off, 20 days (10 injections total); 20d abstinence followed by 1 i.p. injection of 1.5 g/kg EtOH 30 min before trial	Spatial working and reference memory; RAM	Age X Pre-treatment (X Day/Trial)	No acute challenge: Adolescents ↔ Adults; Acute Challenge: EtOH-adolescents ↑ distance traveled during acquisition trial. EtOH pre-treatment ↑ type 1 working memory errors adolescents ↔ adults
White et al., 2000	Male Sprague-Dawley rats; *N* = 14 Adolescents PND 30, *N* = 14 Adults PND 70	i.p. injection of 5 g/kg 16% (v/v) EtOH or saline, 2d on - 2d off, 20 days (10 injections total); 20d abstinence followed by 1 i.p. injection of 1.5 g/kg 16% (v/v) EtOH 30 min before testing	Spatial working memory; RAM	Age X Treatment X Block; Age X Treatment X Delay; Age X Treatment X Day	Acquisition: Adolescents ↔ Adults; Increasing delay period: Adolescents ↔ Adults; Acute challenge: EtOH-Adolescents ↑ errors vs. controls and adults
			Anxiety; EPM	Age X Treatment	No effect of treatment on either age group
Mejia-Toiber et al., 2014	Male Wistar rats; *N* = 50 Adolescents PND 28; *N* = 26 Adults PND 146	3 i.g. injections of 1–5 g/kg 25% (v/v) EtOH, 2d on - 2d off, 26 days (42 injections total); 1–10d abstinence before initial behavioral testing; Adolescents: then 91d abstinence followed by weekly i.p. injection of 0.5, 1, or 2 g/kg EtOH before behavioral session, 19 days; then 18d abstinence followed by 2 i.g. EtOH binge with 1–4 g/kg 25% (v/v) EtOH 6 h apart, 4 days; then 1d abstinence before behavioral testing; Adults: then 80d abstinence followed by weekly i.p. injection of 0.5, 1, or 2 g/kg EtOH before behavioral session, 19 days; then 1d abstinence followed by 2 i.g. EtOH binge with 1–4 g/kg 25% (v/v) EtOH 6 h apart, 4 days; then 1d abstinence before behavioral testing; Note: dosage based on behavioral intoxication score	Delay discounting	Age X Treatment	No effect of chronic exposure on delay discounting performance; Acute EtOH challenges: Adolescents only ↓ preference for large reward regardless of pre-treatment group
			Anxiety; ASR, LPSR	Age X Treatment	EtOH-adolescents ↔ EtOH-adults ↓ LPSR, no treatment effect on ASR; EtOH-adults only ↑ LPSR during withdrawal from 4-day binge
Pickens et al., 2020	Male and female Long Evans rats; *N* = 35 Adolescents PND 27, *N* = 17 Adults (male only) PND 62	2 i.p. injections of 0.875–1.75 g/kg 10% (v/v) EtOH, 1.75–3.5 g/kg 20% (v/v) EtOH, or saline on Mon-Wed-Fri, 6 weeks (36 injections total); 18d abstinence before training	Sign tracking and omission contingency learning	Age X Treatment X Lever X Training day	Sign-tracking: No effect of age or treatment on autoshaping training; EtOH-adolescents vs. adults faster shift to sign-tracking; EtOH ↔ non-exposed groups; Omission contingency learning: low dose EtOH groups vs. high dose and controls slower to decrease responding; no age effects
Slawecki and Ehlers, 2005	Male Sprague-Dawley rats; *N* = 16–17 Adolescents PND 30, *N* = 16–17 Adults PND 61–65	12 h/day, EtOH vapor in sealed chamber, 14 days; 6d abstinence	Anxiety; ASR, PPI	Age X Treatment	PPI: EtOH-adolescents only ↑ vs. controls at 75db, 73db and 76db (non-significant interaction) but not 82db; ASR: EtOH-adolescents ↔ EtOH-adults ↓ magnitude of startle
Conrad and Winder, 2011	Male C57Bl/6J mice; *N* = 14–22 Adolescents PND 28, *N* = 14–20 Adults PND 70–84	i.p. injection of 0.8 g/kg EtOH + 1 mmol/kg pyrazole or 1 mmol/kg pyrazole daily, followed by random exposure (average 3.75 exposures/week) to unpredictable air or EtOH vapor (20.3 ± 0.2 mg/L) for 16 h starting 30 m after injection, 8–10 weeks; 4–6 h abstinence; Note: all animals experiencing chronic social isolation and unpredictable stress	Anxiety; EPM, SIT	Age X Treatment	EPM: EtOH-adolescents ↓ anxiety-like behavior vs. controls and adult groups; SIT: EtOH-adults ↓ anxiety-like behavior vs. controls and adolescent groups
			Neurotransmission; Glu plasticity in BNST	Age X Treatment	Control groups ↑ LTP of N2 after 55 minutes vs. adults; no age differences
Slawecki et al., 2006	Male Sprague-Dawley rats; *N* = 16–22 Adolescents PND 28–30, *N* = 15–22 Adults PND 60–70	12 h/day EtOH vapor in sealed chamber, 14 days; 7–10 h abstinence	Anxiety; L-D box, ASR, PPI	Age X Treatment X Day X PPI	L-D box: EtOH-adults only ↓ transitions, ↑ rearing (indices of mild anxiety); ASR & PPI: No interaction between age and treatment
	Male Sprague-Dawley rats; *N* = 30 Adolescents PND 28–30, *N* = 30 Adults PND 60–70	12 h/day EtOH vapor in sealed chamber, 14 days; 7–10 h abstinence	Brain function; EEG	Age X Treatment X Day	EtOH-adolescents only ↑ vs. controls power 16–32 and 32–50 Hz bands exposure days 2–12 in parietal regions; no treatment effect in FC regions
Slawecki and Roth, 2004	Male Sprague-Dawley rats; *N* = 32 Adolescents PND 31–33, *N* = 32 Adults PND 60–70	12 h/day EtOH vapor in sealed chamber, 5 or 12 days; 7–9 h abstinence	Anxiety; OFT	Age X Treatment X Day	No effect of treatment on anxiety-like behavior
Van Skike et al., 2015	Male Sprague-Dawley rats; *N* = 24 Adolescents PND 28, *N* = 24 Adults PND ~120	i.p. injection of 4 g/kg 20% (v/v) EtOH or saline every other day, 20 days (10 injections total); 24 h or 12d abstinence	Anxiety; ETM	Age, Treatment X Withdrawal time	EtOH-adolescents ↔ EtOH adults ↑ vs. controls anxiety behavior, prolonged withdrawal ↑ anxiety in EtOH groups and adolescent controls
			Neurotransmitters; GABA & NMDA (Glu) receptor protein expression	Age, Treatment X Withdrawal time	No effect of treatment in either age
Morales et al., 2011	Male and female Sprague-Dawley rats; *N* = ? Adolescents PND 24, *N* = ? Adults PND 69	i.p. injection of 2 g/kg 12.6% (v/v) EtOH or saline daily; 10 days; 1d abstinence followed by an i.p. injection of 1 (adults) or 1.25 (adolescents) g/kg EtOH followed by 5 or 25 min abstinence period before behavioral testing	Social activity	Age X Sex X Treatment X Acute Challenge X Injection time	Acute challenge: EtOH-adults ↓ vs. controls social impairment 5 min. post injection; EtOH-adolescents ↓ vs. controls social impairment 25 min. post injection; No acute challenge: adolescents (esp. males) ↑ vs. adults social activity; no interaction between age and treatment
Varlynskaya and Spear, 2007	Male and female Sprague-Dawley rats; *N* = 100 Adolescents PND 27, *N* = 100 Adults PND 62	i.p. injection of 1 g/kg 12.6% (v/v) daily, 7 days; 2d abstinence followed by i.p. injection of 0, 0.25, 0.5, 0.75, or 1 g/kg 12.6% (v/v) EtOH immediately before behavioral testing	Social activity	Age X Sex X Treatment X Acute Challenge	Acute challenge: EtOH-adolescents ↑ acute doses for social facilitation and no social inhibition at high doses compared to control adolescents; EtOH-adults only show ↓ social activity at 1 g/kg; EtOH-adolescents ↑ social preference at 0.5, 0.75, and 1.0 g/kg doses, while EtOH-adults no changes in social preference; No acute challenge: EtOH-adolescents only ↓ vs. controls social preference
Pascual et al., 2009	Male Wistar rats; Adolescents *N* = 40 PND 25, *N* = 40 Adults PND 70	i.p. injection of 3 g/kg 25% (v/v) EtOH or saline, 2d on - 2d off, 14 days (8 injections total); 24 h abstinence	Neurotransmitters; DRD1, DRD2 & NR2B-NMDA (Glu) receptor phosphorylation levels	Age, Treatment	DA: EtOH-adolescents only ↓ vs. controls DRD1 in frontal cortex, DRD2 in FC, striatum, and NAc; Glu: EtOH-adolescents only ↓ vs. controls phosphorylation of NR2B in FC, HC, and NAc
			Neuroplasticity; Histone acetylation in the FC, HC, striatum and NAc	Age, Treatment	EtOH-adolescents only ↑ vs. controls H3 and H4 acetylation in FC and NAc, ↓ striatum, no change in HC
Falco et al., 2009	Male Long Evans rats; *N* = 22 Adolescents PND 28, *N* = 18 Adults PND 80	1 h/day, 10% (v/v) EtOH or water, 18 days; 60d abstinence; Note: all EtOH sessions followed by 30 m delay and 30 m water access	GABAa α1 mRNA expression in BLA	Age, Treatment	EtOH-adults ↓ vs. controls, correlated with GAD67 levels; EtOH-adolescents ↔ adolescent controls
			GAD67 mRNA expression in BLA	Age, Treatment	EtOH-adults ↓ vs. controls, correlated with GABAa α1 levels; EtOH-adolescents ↔ adolescent controls
			CRF mRNA expression in BLA	Age, Treatment	EtOH-adults ↓ vs. controls; EtOH-adolescents ↔ adolescent controls
			NR2A (Glu) mRNA expression in BLA	Age, Treatment	No effect of EtOH in either age
			EtOH consumption	Age X Time	Adolescents ↑ vs. adults day 4–6
Pian et al., 2010	Male Wistar rats; *N* = 42 Adolescents PND 23, *N* = 42 Adults PND 60	14 h/day EtOH vapor in sealed chamber, 14 days; 0 h, 24 h, or 2w abstinence	Neurotransmission; NMDA (Glu) receptor levels in FC and HC	Age, Treatment, Withdrawal	(all vs. age-match controls only) FC: in adults NR1 (0 h ↓ & 24 h ↓), NR2A (0 h ↓ & 24 h ↑) & NR2B (0 h ↓ & 24 h ↓ & 2wk ↑) and in adolescents NR1 (0 h ↓). HC: in adults NR1 (0 h ↓), NR2A (0 h ↑) & NR2B (0 h ↑) and in adolescents NR1 (24 h ↓, & 2wk ↑), NR2A (0 h ↓ & 24 h ↓ & 2wk ↑).
Grobin et al., 2001	Male Sprague-Dawley rats; *N* = ? Adolescents PND 30, *N* = ? Adults PND 90	i.p. injection of 5 g/kg 20% (v/v) EtOH every 120 h, 20 days (5 injections total); 5d, 12d or 33d abstinence	Neurotransmisson; GABAa in neocortex (measured as muscimol-stimulated 36Cl- uptake from tissues samples with and without neurosteroid THDOC)	Age X Treatment	No effects of age or treatment on basal GABAa receptor function; with THDOC neurosteroid: EtOH-adolescents only ↓ potentiation of GABAa with 5 and 12 days of abstinence only present after 33 days of withdrawal
Fleming et al., 2013	Male Sprague-Dawley rats; *N* = ? Adolescents PND 30, *N* = ? Young Adults PND 50, *N* = ? Adults PND 70	i.g. injection of 5 g/kg 35% (v/v) EtOH, 2d on - 2d off, 18 days (10 injections total); 23d abstinence	Neurotransmission; GABAa in HC	Age X Treatment	No significant interaction; post-hoc t-tests: EtOH-adolescents ↓ vs. young adults and adults tonic inhibitory current amplitude & ↑ sensitivity to EtOH exposure on tonic currents
Carerra-Nascimento et al., 2020	Male Swiss mice; *N* = 8–10 Adolescents PND 28–30; *N* = 8–10 Adults PND 68–70	i.p. injection of 2 g/kg 20% (v/v) EtOH or saline daily, 15 days; 5d abstinence followed by i.p. injection of 2 g/kg 20% (v/v) EtOH or saline, sacrificied after 40 m	Neurotransmission; DA and related metabolites	Age X Pretreatment X Challenge	PFC: EtOH-EtOH adolescents ↓ vs. adults and SAL-ETOH-adolescents DA, DOPAC & HVA levels; NAc & striatum: EtOH-EtOH adolescents ↔ adults DA, DOPAC & HVA levels
Vetreno et al., 2014	Male Sprague-Dawley; *N* = ? Adolescents PND 28; *N* = ? Adults PND 70	i.g. injection of 4 g/kg 25% (v/v) EtOH or water every other day, 20 days (10 injections total); 25d abstinence	Neurotransmissionl; levels of ChAT expressing neurons (ChAT + IR) in basal forebrain	Age X Treatment	EtOH-adolescents only ↓ vs. controls
Broadwater et al., 2014	Male Sprague-Dawley rats; *N* = 20 Adolescents PND 28, *N* = 20 Adults PND 70	i.g injection of 4 g/kg EtOH or water every other day, 20 days (11 injections total); 22d abstinence followed by pavlovian tone fear conditioning for separate experiment, sacrificed after 48 h	Neurogenesis in HC and subventricular zone	Age X Treatment	EtOH-adolescents only ↓ vs. controls dentate gyrus neurogenesis via ↑ cell death
Crews et al., 2000	Male Sprague-Dawley rats: *N* = 13 Adolescents PND 25, *N* = 31 Adults PND 80–90	4 i.g. injections of 15% (w/v) EtOH daily (9–10 g/kg daily total), 4 days (16 injections total); Adults: 1 h, 16 h, 72 h, or 168 h abstinence, Adolescents: 1 h abstinence	Neurodegeneration; amino cupric silver staining	Age X Treatment X Withdrawal (in adults)	EtOH-Adolescents ↑ damage vs. adults olfactory and frontal-anterior piriform and perirhinal cortices, EtOH-adults ↑ damage vs. adolescents posterior piriform and perirhinal regions. EtOH-Adolescents ↔ EtOh-adults damage in entorhinal and dentate gyrus
Huang et al., 2012	Male and female C57Bl/6J mice; *N* = 12 Adolescents PND 25, *N* = 12 Adults PND 180	i.p. injection of 3.75 g/kg EtOH daily, 45 days; 1d abstinence	Neurodevelopment; brain mass in cerebral cortex, cerebellum and corpus callosum	Age X Gender X Treatment	EtOH-adolescents only ↓ cerebral cortex mass, EtOH-adults only ↓ corpus callosum length; male ↔ females
Faria et al., 2008	Male Swiss mice; *N* = 10 Adolescents PND 27–28, *N* = 10 Adults PND 57–58	i.p. injection of 2 g/kg 20% (v/v) EtOH or saline daily, 15 days; 7d abstinence followed by i.p. injection of 2 g/kg 20% (v/v) EtOH, sacrified 1 h after	c-Fos expression in PFC, NAc and HC	Age X Treatment	c-Fos in PFC and NAc EtOH-adolescents ↓ vs. adults; in HC EtOH-adults ↓ vs. adolescents
			Egr-1 protein expression in PFC, NAc and HC	Age X Treatment	Egr-1 in PFC, NAc, and HC EtOH-adolescents ↓ vs. adults
Kane et al., 2014	Male and female C57BI/6J mice; *N* = 6 Adolescents PND 35, *N* = 6 Adults PND 84; Note: total *N* = 37 in gene expression, groups not specified	2 i.g. injections of 15% (w/v) EtOH daily (6 g/kg daily total), 10 days (20 total); 1d abstinence	Neuroimmune; gene expression of chemokines, cytokines, and astrocytes	Age X Treatment	EtOH-adults only ↓ chemokine and cytokine gene expression in HC, cerebral cortex, and cerebellum; EtOH-adults ↔ EtOH-adolescents astrocyte expression; EtOH-adults only changed astrocyte morphology in CA1 region
Marshall et al., 2020	Male Sprague-Dawley rats; *N* = 31 Adolescents PND 35, Adults *N* = 44 PND ~70	3 i.g. injections of 5 g/kg 25% (w/v in Vanilla-Ensure Plus ®; initial dose) EtOH or control diet daily (subsequent doses titrated based on behavioral intoxication scores), 2 days (6 injections total) or 4 days (12 injections total) days; No abstinence	Neuroimmune; microglia	Age, Treatment X Duration	EtOH dose needed to reach same intoxication: Adults ↓vs. adol.; EtOH-adults and adolescents ↓ microglia in dentate gyrus, CA fields, and peri-entorhinal cortices; EtOH-Adults and adolescents ↑ microglia dystrophia after 2d and 4d in dentate gyrus and CA fields; 2d ↑ vs. 4d
Slawecki et al., 2005	Male Sprague-Dawley rats; *N* = 17 Adolescents PND 30, *N* = 17 Adults PND 80–90	12/day EtOH vapor, 10 days; 7w abstinence	Levels of neuropeptide-Y, Neurokinines, substance-P, and CRH in HC, FC, HYP, caudate, and amygdala	Age X Treatment	Neuropeptide-Y: EtOH-adults ↑ reduction vs. adolescents in HC only; Neurokinines: EtOH-adults only ↑ caudate levels only; Substance P & CRH: no treatment X age interactions
Rhoads et al., 2012	Male Long Evans and male Sprague-Dawley rats; *N* = ? Adolescents PND 25–28, *N* = ? Adults PND > 74	24 h/day, 6.7% (v/v) EtOH liquid diet or EtOH-free liquid diet, 3 weeks; no abstinence	EtOH consumption	Age X Strain	Adolescents ↑ vs. adults in both strains
			Brain catalase levels	Age X Strain	No effects of age or treatment in either strain
Galaj et al., 2020	Male Sprague-Dawley rats; *N* = ? Adolescents PND 28, *N* = ? Adults PND 70; Note: total *N* = 124, groups not specified	i.g. injection of 4 g/kg 25% (v/v) EtOH or water daily, 3d on - 2d off, 20 days (12 injections total); 2d or 21d abstinence	Prelimbic cortex pyramidal neuron functioning	Age X Treatment X Abstinence Period	2d abstinence: EtOH-adolescents only ↓ sEPSC amplitude in early withdrawal vs. controls; no treatment effect on frequency; EtOH-adolescents ↑ thin spine ratio in PrL-L5 neurons; 21d abstinence: EtOH-adolescents ↓ sEPSC frequency and amplitude in PrL-L5 neurons, while EtOH-adults ↑; EtOH-adolescents ↓ total spine density and non-thin spine density, while EtOH-adults ↑; no treatment effects in PrL-L2
Li et al., 2013	Male Sprague-Dawley rats; *N* = 8–23 Adolescents PND 30, *N* = 8–24 Adults PND 70	i.g. injection of 5 g/kg 35% (w/v in 0.9% saline) EtOH or saline, 2d on - 2d off, 20 days (10 injections total); 20d abstinence	Voltage-gated A-type potassium channel functioning in CA1 interneurons	Age, Treatment	Mean peak amplitude: EtOH-adolescents and adults ↓ vs. controls; Density: EtOH-adolescents only ↓ vs. controls; voltage-dependent steady-state activation: EtOH-adolescents only ↑ vs. control; voltage-dependent steady-state inactivation: EtOH-adolescents only ↑ vs. controls required depolarization for activation and ↑ slow decay time; EtOH-adults ↓ slow decay time

Studies are listed in order of appearance in the text within the voluntary and forced sections. Only analyses assessing differences between adolescents and adults in the effect of alcohol on brain or cognitive outcomes are listed.

*ASR* acoustic startle response, *BDNF* brain-derived neurotropic factor, *BLA* basolateral amygdala, *BNST* bed nucleus of the stria terminalis, *CA* Cornu ammonis, *CaMK* calcium-dependent protein kinase, *Cdk* cyclin-dependent kinase, *ChAT* choline acetyltransferase, *CPA* conditioned place aversion, *CPP* conditioned place preference, *CRH* corticotropin-release hormone, *CTA* Conditioned taste aversion, *DA* Dopamine, *DID* Drinking in the dark, *DRD* dopamine receptor, *Egr* early growth response protein, *EPM* elevated plus maze, *ETM* elevated T-maze, *EtOH* ethanol, *FC* frontal cortex, *GABA* gamma aminobutyric acid, *Glu* glutamate, *HAT* histone acetyltransferase, *HC* hippocampus*, HDAC* histone deacetylase, *HYP* hypothalamus, *i.g.* Intragastric, *i.p.* INtraperitoneal, *L-D box* light-dark box, *LPSR* light-potentiated startle response, *LTP* long-term potentiation, *MBT* marble burying test, *mGlu* metabolic glutamate receptor, *MWM* Morris water maze, *NAc* nucleus accumbens, *NGF* nerve growth factor, *NMDA* N-methyl-D-asparaginezuur, *NOR* novel object recognition, *NOT* novel object test, *OFT* open field test, *PFC* prefrontal cortex, *PKC* protein kinase C, *PND* postnatal day, *PPI* prepulse inhibition, *RAM* radial arm maze, *SBM* sand box maze, *SIT* social interaction test, *SOPC* second-order place conditioning, *v/v* volume/volume, *?* Unknown.

**Table 2:** Characteristics and findings of human studies on age-related differences on the effect of alcohol on cognition and the brain.

Author	Outcomes	Sample (*N*, age)	Measurement of Alcohol Use	Design	Result	Quality of evidence
Müller-Oehring et al., 2018	Brain function: resting state functional connectivity in default mode, executive control, salience, emotion, and reward networks	12 to 21 years old; *N* = 581 no/low drinkers *N* = 117 heavier drinkers	No/low drinking group: lifetime drinks criteria - age 12–15 <6, age 16 <12, age 17 <24, age 18–21 <52; heavier drinkers: exceed thresholds, *N* = 9 with DSM-IV Alcohol Abuse, *N* = 1 Alcohol Dependence	Seed-based correlation analysis, alcohol use based group analysis with age and sex as covariates; Correlations between brain function and neuropsychological performance	*Default mode*: No alcohol effects; *Executive control*: SFG-Insula connectivity ↑ with age in no/low drinkers only; *Salience*: No alcohol effects; *Emotion*: Amygdala-medial parietal synchrony ↓ in heavier drinkers vs. no/low group; age-related default mode and emotional network desynchronization neg. correlated with episodic memory performance in heavier drinking group; *Reward*: No alcohol effects	Moderate
McAteer et al., 2018	Alcohol attentional bias	*N* = 42 Early Adolescents 12–13 yrs *N* = 42 Late Adolescents 16–17 yrs *N* = 55 Young Adults 18–21 yrs	Stratified based on AUDIT; Heavy drinkers: ≥9, light drinkers = 1–8, non-drinkers = 0	Age (late adolescent, young adults) X Drinking (heavy, light drinkers); Non-drinkers analyzed separately by Age	No effect of age in non-drinkers; Heavy drinkers ↑ fixation on alcohol cues vs. light drinkers regardless of age	Weak
Rooke and Hine, 2011	Alcohol-related implicit memory associations; Explicit alcohol outcome expectancies	*N* = 138 Adolescents 13–19 yrs, *N* = 138 Adults 29–73 yrs; Teen-parent dyads	Binge-drinking: a composite score based on self-reported avg. # of drinks per occasion and frequency of 4+ drink occasions	Multiple regression; DV = binge-drinking, IV = Age, Moderators: implicit, explicit cognitions; Control variables: Sex	Adolescents ↑ binge drinking, alcohol memory associations, and expected benefits vs. adults; Adolescents ↔ Adults expected costs; Memory association, expected benefits ↑ predictor of binge-drinking in adolescents vs. adults	Weak
Cousijn et al., 2020	Craving, alcohol attentional bias, alcohol approach bias, drinking motives, impulsivity, interference control, decision-making (risky), working memory	*N* = 45 Adolescents 16–17 yrs, *N* = 45 Adults 30–35 yrs	1x/month to daily drinkers in each age group (matched); Alcohol-related problems severity: AUDIT score; number of drinking days/month; number of drinks/episode	Multiple regression; DV = AUDIT problems, monthly alcohol use, drinks per drinking episode; IVs: Cognitive and motivational outcomes; Moderator: age	Adolescents ↔ adults monthly alcohol use; Adolescents ↑ drinks per episode, ↓ drinking days per month vs. adults; Adolescents ↑ impulsivity, enhancement drinking motives; Adolescents ↔ adults attentional bias to alcohol; no approach bias in either group; Adolescents ↔ risky decision-making, interference control, working memory; only impulsivity and social, coping, and enhancement motives correlated with alcohol use; age moderates correlation between social drinking motives and AUDIT with a stronger positive association in adolescents vs. adults	Weak

Only analyses assessing differences between adolescents and adults in the effect of alcohol on brain or cognitive outcomes are listed. Quality of evidence: strong = longitudinal design comparing adolescent and adults while accounting for relevant covariates; moderate = longitudinal design comparing adolescents and adults without adjusting for relevant covariates or cross-sectional designs with matched groups that considered relevant covariates; weak = cross-sectional design without matched adolescent and adult groups and/or did not adjust for relevant covariates.

*AUDIT* Alcohol Use Disorder Identification Test, *IV* Independent variable, *DV* Dependent variable.

### Animal studies

#### Cognitive outcomes

##### Learning and memory

Human evidence clearly suggests that alcohol is related to learning and memory impairments, both during intoxication [44] and after sustained heavy use and dependence [45, 46]. Paradigms that assess learning and memory provide insight into the negative consequences of alcohol consumption on brain functioning, as well as the processes underlying the development and maintenance of learned addictive behaviors.

- *Conditioned alcohol aversion or preference:* Lower sensitivity to alcohol’s aversive effects (e.g., nausea, drowsiness, motor incoordination) but higher sensitivity to alcohol’s rewarding effects has been hypothesized to underlie the higher levels of alcohol use, especially binge-like behavior, in adolescents compared to adults [47]. Several conditioning paradigms have been developed to assess the aversive and motivational effects of alcohol exposure.
- The conditioned taste aversion (CTA) paradigm is widely used to measure perceived aversiveness of alcohol in animals. Repeated high-dose ethanol injections are paired with a conditioned stimulus (CS, e.g., a saccharin or NaCL solution). The reduction in CS consumption after conditioning is used as an index of alcohol aversion. Two studies examined CTA in mice [48, 49] and two in rats [50, 51]. Three of the four studies found age-related differences. In all three studies using a standard CTA paradigm, adolescents required a higher ethanol dosage to develop aversion compared to adults [48–50]. Using a similar second-order conditioning (SOC) paradigm pairing high doses of ethanol (3.0 g/kg) with sucrose (CS), both adolescent and adult rats developed equal aversion to the testing compartment paired with ethanol [51].
- Overall, three studies found support for lower sensitivity to alcohol’s aversive effects in adolescents, whereas one observed no differences. Future research should employ intragastric as opposed intraperitoneal exposure to better mimic human binge-like drinking in order to increase the translational value of the findings.
- To measure differences in alcohol’s motivational value, conditioned place preference (CPP) paradigms have been used. This involves repeated pairings of ethanol injections with one compartment and saline injections with another compartment of the testing apparatus. On test days, CPP is assessed by measuring how long the animal stays in the compartment paired with ethanol relative to saline injections. Four studies examined CPP, with two studies observing age-related differences [52–55]. In the only mouse study, history of chronic ethanol exposure during adolescence (2.0 g/kg for 15 days) but not adulthood [52] led to increased CPP after brief abstinence (5 days) before the conditioning procedure (2.0 g/kg, four doses over 8 days). This suggests that early ethanol exposure increases alcohol’s rewarding properties later on. However, two rat studies did not observe either preference or aversion in either age when using lower ethanol doses and a shorter exposure period (0.5 and 1.0 g/kg for 8 days) [53], nor when using higher doses and intermittent exposure (3.0 g/kg, 2 days on, 2 days off schedule) [55]. Next to species and exposure-specific factors, environmental factors also play a role [54], with adolescents raised in environmentally enriched conditions demonstrating CPP (2 g/kg) while adolescents raised in standard conditions did not. In contrast, CPP was insensitive to rearing conditions in adults with both enriched and standard-housed rats showing similar levels of CPP.
- Overall, there is inconsistent evidence for age-related differences in the motivational value of ethanol. One study found support for increased sensitivity to the rewarding effects of ethanol in adolescents, whereas one found support for adults being more sensitive and two observed no differences.
- *Fear conditioning and retention:* Pavlovian fear conditioning paradigms are used to investigate associative learning and memory in animals. These paradigms are relevant for addiction because fear and drug-seeking behavior are considered conditioned responses with overlapping neural mechanisms [56]. Rodents are administered an unconditioned stimulus (US; e.g., foot shock) in the presence of a conditioned stimulus (CS; unique context or cue). Conditioned responses (CR; e.g., freezing behavior) are then measured in the presence of the CS without the US as a measure of fear retention. Contextual fear conditioning is linked to hippocampus and amygdala functioning and discrete cue-based (e.g., tone) fear is linked to amygdala functioning. [57–59], and fear extinction involves medial PFC functioning [60]. Five studies investigated fear conditioning, four in rats [61–64] and one in mice [65].
- Only one of the four studies observed age-related differences in tone fear conditioning. Bergstrom et al. [61] found evidence for impaired tone fear conditioning in male and female alcohol-exposed (18d) adolescent compared to adult rats after extended abstinence (30d). However, adolescent rats consumed more ethanol during the one-hour access period than adults, which may explain the observed age differences in fear tone conditioning. Small but significant sex differences in consumption also emerged in the adolescent group, with males showing more persistent impairment across the test sessions compared to females, despite adolescent females consuming more ethanol than males. In contrast, three studies found no evidence of impaired tone fear conditioning in either age group after chronic alcohol exposure (4 g/kg, every other day for 20d) and extended abstinence [62, 63] (22d), [64].
- Two of the three studies observed age-related differences in contextual fear conditioning [62–64]. In two studies with similar exposure paradigms, only adolescents exposed to chronic high dosages of ethanol (4 g/kg) showed disrupted contextual fear conditioning after extended abstinence (22d) [62, 63]. Importantly, differences disappeared when the context was also paired with a tone, which is suggestive of a potential disruption in hippocampal-linked contextual fear conditioning specifically [64]. Furthermore, there may be distinct vulnerability periods during adolescence as contextual fear retention was disrupted after chronic alcohol exposure (4 g/kg, every other day for 20d) during early-mid adolescence but not late adolescence [62]. In the only study to combine chronic exposure and acute ethanol challenges, contextual conditioning was impaired by the acute challenge (1 g/kg) but there was no effect of pre-exposure history in either age group (4 g/kg, every other day for 20d) [63].
- Only one study examined fear extinction, and found no effect of ethanol exposure (4/kg, every other day for 20d) on extinction after tone conditioning. However, adults had higher levels of contextual fear extinction compared to mid-adolescents while late adolescents performed similar to adults [62]. Moreover, looking at binge-like exposure in mice (three binges, 3d abstinence), Lacaille et al. [65] showed comparable impairments in long-term fear memory in adolescents and adults during a passive avoidance task in which one compartment of the testing apparatus was paired with a foot shock once and avoidance of this chamber after a 24 h delay was measured.
- In sum, there is limited but fairly consistent evidence for adolescent-specific impairments in hippocampal-linked contextual fear conditioning across two rat studies, while no age differences emerged in context-based fear retention in one study of mice. In contrast, only one of the four studies found evidence of impaired tone fear conditioning in adolescents (that also consumed more alcohol), with most finding no effect of alcohol on tone fear conditioning regardless of age. With only one study examining medial PFC-linked fear extinction, no strong conclusions can be drawn, but initial evidence suggests context-based fear extinction may be diminished in mid-adolescents compared to adults and late adolescents. Research on age-related differences on the effect of alcohol on longer-term fear memory is largely missing.
- *Spatial learning and memory:* The Morris Water Maze (MWM) is commonly used to test spatial learning and memory in rodents. Across trials, time to find the hidden platform in a round swimming pool is used as a measure of spatial learning. Spatial memory can be tested by removing the platform and measuring the time the animal spends in the quadrant where the escape used to be. The sand box maze (SBM) is a similar paradigm in which animals need to locate a buried appetitive reinforcer.
- Six rat studies examined spatial learning and memory using these paradigms. Three of the six studies observed age-related differences. Four examined the effects of repeated ethanol challenges 30 minutes prior to MWM training, showing mixed results [30, 66–68]. While one found ethanol-induced spatial learning impairments in adolescents only (1.0 and 2.0 g/kg doses) [66], another found no age-related differences, with both age groups showing impairments after moderate doses (2.5 g/kg) and enhancements in learning after very low doses (0.5 g/kg) [67]. Sircar and Sircar [68] also found evidence of ethanol-induced spatial learning and memory impairments in both ages (2.0 g/kg). However, memory impairments recovered after extended abstinence (25d) in adults only. Importantly, MWM findings could be related to thigmotaxis, an anxiety-related tendency to stay close to the walls of the maze. Developmental differences in stress sensitivity may potentially confound ethanol-related age effects in these paradigms. Using the less stress-inducing SBM, adults showed greater impairments in spatial learning compared to adolescents after 1.5 g/kg ethanol doses 30 min prior to training [30].
- Two studies examined the effects of chronic ethanol exposure prior to training with or without acute challenges [69, 70]. Matthews et al. [70] looked at the effect of 20 days binge-like (every other day) pre-exposure and found no effect on spatial learning in either age following an extended abstinence period (i.e., 6–8 weeks). Swartzwelder et al. [69] examined effects of 5-day ethanol pre-exposure with and without ethanol challenges before MWM training. Ethanol challenges (2.0 g/kg) impaired learning in both age groups regardless of pre-exposure history. Thigmotaxis was also increased in both age groups after acute challenges while pre-exposure increased it in adults only.
- In sum, evidence for impaired spatial learning and memory after acute challenges is mixed across six studies. Two studies found support for ethanol having a larger impact in adolescents compared to adults, whereas one study found the opposite and three studies did not observe any differences. Differences in ethanol doses stress responses may partially explain the discrepancies across studies. Importantly, given the sparsity of studies addressing the effects of long-term and voluntary ethanol exposure, no conclusion can be drawn about the impact of age on the relation between chronic alcohol exposure and spatial learning and memory.
- *Non-spatial learning and memory:* Non-spatial learning can also be assessed in the MWM and SBM by marking the target location with a pole and moving it across trials, measuring time and distances traveled to locate the target. By assessing non-spatial learning as well, studies can determine whether learning is more generally impaired by ethanol or whether it is specific to hippocampal-dependent spatial learning processes. A total of six studies assessed facets of non-spatial learning and memory. Two of the six studies observed age-related differences.
- In the four studies that examined non-spatial memory using the MWM or SBM in rats, none found an effect of alcohol regardless of dose, duration, or abstinence period in either age group [30, 66, 67, 70]. Two other studies examined other facets of non-spatial memory in rats [65, 71]. Galaj et al. [71] used an incentive learning paradigm to examine conditioned reward responses and approach behavior towards alcohol after chronic intermittent ethanol (CIE; 4 g/kg; 3d on, 2d off) exposure to mimic binge drinking. To examine reward-related learning and approach behavior, a CS (light) was paired with food pellets and approach behavior to CS only presentation and responses to a lever producing the CS were measured. In both adolescents and adults, the ethanol-exposed rats showed impaired reward-related learning after both short (2d) and extended (21d) abstinence. No effect of alcohol on conditioned approach behavior was observed in either age group during acute (2d) or extended (21d) abstinence. Using a novel object recognition test in mice, Lacaille et al. [65] assessed non-spatial recognition memory by replacing a familiar object with a novel object in the testing environment. Explorative behavior of the new object was used as an index of recognition. After chronic binge-like exposure (three injections daily at 2 h intervals) and limited abstinence (4d), only adolescents showed reduced object recognition.
- Across facets of non-spatial memory, there is little evidence for age-related differences in the effect of chronic alcohol, with four of the six studies finding no age differences. For memory of visually cued target locations in the MWM and SBM paradigms, alcohol does not alter performance in either age. Also, both adolescents and adults appear similarly vulnerable to alcohol-induced impairments in reward-related learning based on the one study. Only in the domain of object memory did any age-related differences emerge, with adolescents and not adults showing reduced novel object recognition after binge-like alcohol exposure in one study. However, more research into object recognition memory and reward-related learning and memory is needed to draw strong conclusions in these domains.

##### Executive function and higher-order cognition

Executive functions are a domain of cognitive processes underlying higher-order cognitive functions such as goal-directed behavior. Executive functions can include but are not limited to working memory, attentional processes, cognitive flexibility, and impulse control or inhibition [72]. A core feature of AUD is the transition from goal-directed alcohol use to habitual, uncontrolled alcohol use. Impaired executive functioning, linked to PFC dysfunction [73], is assumed to be both a risk factor and consequence of chronic alcohol use. A meta-analysis of 62 studies highlighted widespread impairments in executive functioning in individuals with AUD that persisted even after 1-year of abstinence [46]. Thirteen studies examined facets of executive functioning and higher-order cognition, specifically in the domains of working memory, attentional processes, cognitive flexibility, impulsivity in decision-making, and goal-directed behavior [65, 74–83].

- *Working memory:* Working memory refers to the limited capacity system for temporarily storing and manipulating information, which is necessary for reasoning and decision-making [84]. In the Radial Arm Maze test (RAM) [85], some of the equally spaced arms (typically eight) around a circular platform contain a food reward for animals to find. Spatial working memory is measured by recording the number of revisits to previously visited arms (i.e., working memory error) and first entries into unbaited arms (i.e., reference memory). Alternatively, the hippocampus mediated [86] spontaneous tendency to alternate arms can be used as a measure of spatial working memory. In this case, revisiting an arm in back-to-back trials in close temporal succession is interpreted as a working memory error. Five studies examined the effects of chronic ethanol exposure on spatial working memory [65, 75, 79, 80, 83]. One of the five studies observed age-related differences.
- Chronic binge-like alcohol exposure had no effects on spontaneous alterations after prolonged abstinence (2d on, 2d off; 3 weeks abstinence) [79, 80] in rats or limited abstinence (three injections daily at 2 h intervals; 24 h abstinence) [65] in mice, nor on RAM performance in rats (2d on, 2d off) [75, 83]. However, acute ethanol challenges (1.5 g/kg) after chronic binge-like exposure (2d on, 2d off) resulted in RAM test impairments in both age groups in rats [75, 83], with some evidence for increased working memory errors in adolescents [83].
- In sum, there is little evidence for impairments in working memory function in rats after chronic ethanol exposure, with four of the five studies observing no difference between age groups. While acute intoxication impairs working memory function in both ages, there is evidence from only one study that adolescents may make more working memory errors.
- *Attentional processes:* Attentional processing refers to the selection of information that gains access to working memory [87]. PPI is a pre-attentional cognitive function which provides an index of sensorimotor gating and measures the ability of a lower intensity sensory stimulus to reduce the magnitude of response to a more intense stimulus presented closely afterward. Reduced sensorimotor gating (reduced PPI) can disrupt information processing and thereby impair cognitive function, while enhanced sensorimotor gating (enhanced PPI) may reflect behavioral inflexibility [88]. For example, lesions in the medial PFC produce both behavioral inflexibility and enhancements in PPI in rats. Two studies assessed attentional processes by measuring prepulse inhibition (PPI) in rats [82, 89]. One study observed age-related differences and one did not.
- Slawecki and Ehlers [82] observed age-related differences in sensorimotor gating following ethanol vapor exposure (2w) and brief abstinence (6d), with adolescents showing enhanced PPI at some decibels reflective of behavioral inflexibility, while adults did not exhibit PPI at any of the intensities tested. Slawecki et al. [89] did not observe any age-related differences in PPI during the acute phase of ethanol withdrawal (7–10 h abstinence) during a period of chronic ethanol exposure (14d).
- In sum, there is limited and mixed evidence from two studies of age-related differences in the pre-attentional process of sensorimotor gating. Only one study found support for adolescent sensitivity to ethanol effects.
- *Cognitive flexibility:* Cognitive flexibility refers to the ability to update information based on environmental factors r changing goals in order to adaptively guide decision-making and is linked to the inability to reduce or abstain from drinking [90]. Three studies examined facets of cognitive and behavioral flexibility [79–81]. Two of the three studies observed age-related differences.
- In two rat studies, cognitive flexibility was assessed using reversal learning paradigms [79, 80]. In the reversal learning paradigm, rats were trained on simple (e.g., visual cue) and more complex discriminations (e.g., visual + scent cue) between rewarded and non-rewarded bowls. After learning the discriminants, the rewards were reversed. Ethanol exposure reduced flexibility in both adolescents and adults for simple discriminations in both studies. Age-related differences emerged for the more complex discriminations in one study, with only adults showing reduced flexibility after prolonged abstinence (21d) following binge-like exposure (5 g/kg, 2d on, 2d off) [79]. In contrast, both age groups showed reduced flexibility for complex discrimination in the other study after prolonged abstinence (21d) despite adolescents consuming more ethanol orally than adults during the 28 week exposure [80].
- In another study, Labots et al. [81] used a conditioned suppression of alcohol-seeking task after two months of voluntary ethanol consumption (2 months) in rats to examine flexibility around alcohol-seeking behavior. After stratifying the age groups based on levels of ethanol consumption, medium- and high-consuming, adolescents showed higher levels of conditioned suppression compared to similarly drinking adults, indicating greater behavioral flexibility and control over alcohol-seeking in adolescents after chronic voluntary exposure.
- Overall, there is limited evidence for adolescent resilience to the effects of chronic alcohol on cognitive flexibility. Two studies found support for adolescent resilience to ethanol’s effect on behavioral flexibility, whereas another study found no differences between adolescents and adults.
- *Impulsivity:* Impulsivity is a multi-faceted behavioral trait that encompasses impaired response inhibition, preference for an immediate reward over a larger but delayed reward, and premature expression of behaviors which may be maladaptive or in conflict with conscious goals. Impulsivity is a risk-factor for the development of addiction and may also be a consequence of sustained substance use [35]. Pharmacological evidence points towards overlapping neuronal mechanisms in impulsivity and addictive behavior, particularly within the mesolimbic dopamine system [91]. Two studies examined impulsive decision-making behavior in rats [74, 78]. Both studies observed age-related differences.
- One study examined impulsive behavior using a delay-discounting task in which choices are made between immediate small rewards and larger delayed rewards [78]. Regardless of age, chronic intermittent exposure (2d on, 2d off) had no effect on choice behavior in non-intoxicated rats. Following acute challenges, adolescents but not adults demonstrated a reduced preference for the large reward regardless of ethanol exposure history, reflecting a general adolescent-specific heightened impulsivity during intoxication. Another study examined decision-making under risk conditions using an instrumental training and probability-discounting task [74]. After prolonged abstinence (20d), rats were trained to press two levers for sucrose rewards and were concurrently trained to choose between two levers with different associated probabilities of reward and reward size, creating a choice between a certain, small reward and an uncertain, large reward (i.e., riskier choice). Ethanol consumption was voluntary and while adolescents initially consumed more ethanol than adults at the beginning of the exposure period, the total amount of consumption was similar by the end of the exposure period. Only adolescents showed increased risky and sub-optimal decision-making compared to age-matched controls, while adults performed similarly to controls.
- In sum, both studies found support for ethanol having a larger impact on adolescent compared to adults on impulsive behavior.
- *Goal-directed behavior:* Goal-directed behavior refers to when actions are sensitive to both the outcome value (goal) and contingency between the behavior and the outcome [92]. Two studies used a sign-tracking and omission contingency learning paradigm to examine goal-directed versus habitual behavior [76, 77]. One study observed age-related differences and the other did not. Sign tracking refers to tasks where a cue predicts a reward, but no response is needed for the reward to be delivered. Despite this, after repeated pairings of the cue and reward, animals and humans may respond (e.g., via a lever) when the cue is presented anyway, and even when no reward is known to be available. Sign-directed behavior is considered habitual and has been proposed to underlie the lack of control of alcohol use in addiction [93]. In humans, sign-tracking behavior is difficult to differentiate from goal-directed behavior based on only the observable behavior, i.e., seeing a cue such as a favorite drink or bar and then having a drink [94]. In the context of alcohol use, *reflexively* having a drink when seeing an item that is often associated with the rewarding effects of alcohol (e.g., wine glass, bar, smell of alcohol) despite not consciously desiring the alcohol ‘reward’ is an example of how habitual behavior (possibly driven by sign-tracking) can initiate the behavior as opposed to an intentional goal [93]. Omission contingency refers to a 2nd phase after sign-tracking when the response is punished and the behavior must be inhibited to avoid punishment. After both forced and voluntary ethanol exposure (6w), no alterations to sign-tracking behavior were observed in adolescent and adult rats [76, 77]. One study did observe an age-related difference in omission contingency learning, with adolescents performing better than adults after chronic voluntary ethanol exposure [77]. This preliminarily suggests that adolescents may be more capable of adapting their behavior to avoid punishment compared to adults after chronic use. However, before behavioral testing began, adolescent rats were abstinent for 17 days, while adults were only abstinence for 10 days which may have influenced the results.
- In summary, one study found support for adolescents being less sensitive to ethanol effects on goal-directed behavior compared to adults, whereas one study found no effect of ethanol in either age group.
- Across the domains of executive function, there is some evidence that adolescents may be more vulnerable to impairments in certain executive and higher-order cognitive functions following chronic alcohol exposure, with increased risky decision-making after prolonged abstinence [74], impulsivity during intoxication [78], and reduced working memory function during intoxication after chronic exposure. In contrast, animals exposed to alcohol during adolescence may better retain cognitive flexibility [77, 79] and are better able to regain control over alcohol-seeking in adulthood [81].

##### Other behavioral outcomes

- *Anxiety*: AUD is highly comorbid with anxiety disorders [95], especially in adolescence [96]. While anxiety is not strictly a cognitive outcome, it is related to altered cognitive functioning [97, 98]. Many studies assessing the effects of ethanol on the rodent brain and cognition also include anxiety-related measures. Multiple paradigms have been developed to elicit behaviors thought to reflect anxiety in rodents (e.g., rearing, startle, avoidance, etc.). In the open field test (OFT), anxiety is indexed as the tendency to stay close to perimeter walls as animals have a natural aversion to brightly lit open spaces [99]. In the elevated plus maze paradigm, rodents are placed at the center of an elevated four-arm maze with two open arms two closed arms [100]. The open arms elicit unconditioned fear of heights/open spaces and the closed arms elicit the proclivity for enclosed, dark spaces. Anxiety is indexed as entries/duration of time in open vs. closed arms, as well as rearing, freezing, or other postural indices of anxiety. In startle paradigms, the startle response is a defensive mechanism reflecting anxiety which follows a sudden, unpredictable stimulus (e.g., tones, light) [101]. In light-dark box paradigms, anxiety is elicited using a testing apparatus with a light and dark compartment, relying on the conflict between natural aversions to well-lit spaces and the tendency to explore new areas. Percentage of time spent in the light compartment, latency to return to the dark compartment, movement between compartments (transitions), and rearing-behavior are measured as indices of anxiety [102]. Anxiety can also be assessed using a social interaction test with an unfamiliar partner, with approach and avoidance behaviors measured to index anxiety [103]. In the novel object test (NOT) [104], anxiety is elicited by the introduction of a new object in the rodent’s environment. The amount of contacts and time spent in contact with the object is used as an index of anxiety. Similarly, in the marble-burying test (MBT), novel marbles are placed in an environment and the amount of defensive burying of the objects is used as an index of anxiety [105].
- Eleven studies examined anxiety-like behavior in rodents with mixed results across paradigms [70, 78, 82, 83, 89, 106–111]. Overall, five of the eleven studies observed age-related differences.
- Two studies used the OFT, finding no effects of voluntary (2w, 4 h/day access) or forced (12/day vapor) ethanol exposure on anxiety-like behavior in adolescents or adult rats during withdrawal (7–9 h) [110] or after a brief abstinence period (4 days) [107]. One study used both the MBT and NOT after voluntary ethanol consumption (2 h/d for 2 weeks; no abstinence) and observed higher anxiety in ethanol-exposed adults and reduced anxiety in ethanol-exposed adolescents compared to controls as indexed by marble burying [106]. However, no age effects were observed in response to a novel object, with reduced interaction with the novel object in both age groups after chronic exposure.
- Four studies used the elevated maze paradigm with mixed results. Only one study observed age-related differences in mice after chronic exposure (8–10w vapor) [109]. Adolescents showed reduced anxiety compared to adults during the acute withdrawal period, but all mice were kept under chronic social isolation and unpredictable stress conditions, which may have affected the results. Two studies in rats found no effect of intermittent (1 g/kg) or binge-like (5 g/kg) exposure in either age group after short (24 h) [70] or sustained abstinence (20d) [83]. A third study observed heightened anxiety in both age groups after intermittent exposure (4 g/kg), with anxiety increasing with prolonged abstinence periods (24 h to 12d) [108].
- Three rat studies used a startle paradigm to assess anxiety. Two observed reduced acoustic startle responses after ethanol exposure (12 h/d vapor) in both age groups during acute withdrawal periods (7–10 h) and following more sustained abstinence (6d) [82, 89]. In the other study, light-potentiated startle was also reduced in both ages during days 1–10 of withdrawal after binge-like exposure (2d on, 2d off), but age-related differences emerged when the rats were re-exposed via a 4-day binge (1–4/kg). Then, only adults showed higher levels of light-potentiated startle compared to controls [78], suggesting that ethanol pre-exposure increases anxiety in adults but not adolescents when re-exposed to ethanol after withdrawal.
- Two studies used the light-dark box paradigm with mixed results [89, 111]. Only adult rats showed increased mild anxiety-like behaviors during early withdrawal (7–10 h) after chronic vapor exposure 12 h/d) [89]. In contrast, no age-related differences emerged after voluntary ethanol consumption (18 h/d access; 3d/w for 6 weeks), with male mice showing less anxiety-like behavior in both ages [111]. In contrast, the one study using the social interaction test observed reduced anxiety in adult mice compared to both adolescents and age-matched controls during early withdrawal (4–6 h) after chronic, unpredictable vapor exposure [109].
- In summary, there is inconsistent evidence for age-related differences in the effect of chronic ethanol exposure on anxiety outcomes in rodents. The substantial differences across studies in how anxiety was elicited and measured make it challenging to draw strong conclusions. In the five studies that found age-related differences, adults tend to show higher levels of anxiety, particularly during early withdrawal; however, the opposite was found in the one study examining anxiety in social interactions. Six studies did not observe any age-related differences. Overall, adolescents may be less sensitive to the anxiety-inducing effects of chronic alcohol exposure.
- *Social behavior:* Two studies were identified that examined the effects of chronic ethanol exposure on social behavior in rats [112, 113], with both observing age-related differences. After chronic exposure (1 g/kg, 7d), followed by a brief abstinence period (24–48 h), one study found a decrease in social preference in adolescents only [112], while the other study found no ethanol-related effects on social behavior (2 g/kg, 10d) [113]. After acute challenges, age and treatment interactions emerged in both studies, but the directions of the results are inconsistent. In the first study, adolescents showed increased social preference, as indexed by the number of cross-overs between compartments toward and away from a peer, across multiple acute doses (0.5–1.0 g/kg) administered immediately before testing, while adults showed no changes in social preference [112]. In contrast, Morales et al. [113] found evidence for age-related temporal differences in social activity after acute challenge, with adults showing decreased social impairment five minutes post injection (1 g/kg) and adolescents (1.25 g/kg) after 25 min compared to age-matched controls.
- The findings from these two studies paint a complicated and inconsistent picture of the effects of ethanol on social behavior in adults and adolescents warranting further research. One study found support for a larger effect of chronic ethanol on adolescent social behavior compared to adults, while the other did not observe effects of ethanol in either group. One study found support for a larger effect of chronic plus acute ethanol intoxication on social behavior, with the opposite observed in the other.

### Brain outcomes

#### Neurotransmitter systems

##### Glutamate

Glutamate is the brain’s main excitatory neurotransmitter and plays a crucial role in synaptic plasticity (i.e., experience-related strengthening or weakening of synaptic connections). Glutamatergic transmission plays an important role in the formation and maintenance of addictive behaviors and the nucleus accumbens (NAc) is considered an important hub in this, receiving glutamatergic input from cortical-limbic areas and dopaminergic input from the midbrain [114]. Seven studies investigated glutamate functioning in regions of the brain [106–109, 115–118]. Four of the seven studies observed age-related differences.

Three studies investigated glutamate-related processes in the NAc [106, 107, 118]. Two weeks of voluntary binge drinking (4-h access, no abstinence) did not affect expression of calcium-dependent kinase II alpha (CaMKIIα) and the AMPA receptor GluA1 subunit in the NAc of mice [107]. In contrast, Lee et al. [106] showed that voluntary binge drinking (2-h access, no abstinence) increased mGlu1, mGlu5, and GluN2b expression in the shell of the NAc, as well as PKCε and CAMKII in the core of the NAc in adult mice only. In rats, Pascual et al. [118] showed reduced NR2B phosphorylation in the NAc of adolescents only after two weeks of chronic intermittent ethanol exposure; an effect that also lasted until 24 h after end of exposure. This indicates that adolescents might be less affected by the effects of ethanol on NAc-related glutamatergic neurotransmission than adults. This may in turn mediate decreased withdrawal symptoms and potentially facilitate increased drinking [106].

Two studies investigated glutamate-related processes in the (basolateral) amygdala [107, 116]. In mice, Agoglia et al. [107] showed decreased CaMKIIα phosphorylation in adolescents, but increased GluA1 expression in adults after two weeks of voluntary binge drinking (4-h access, no abstinence). Also, drug-induced AMPAR activation resulted in increased binge drinking in adolescents but decreased binge drinking in adults, highlighting the potential importance of glutamatergic signaling in age-related differences in alcohol consumption. However, Falco et al. [116] reported no difference in NR2A mRNA levels in the basolateral amygdala for either age group after 60-day abstinence.

Alcohol’s effects on frontal cortex functioning is thought to be mediated by alterations in NMDA receptor subunit expression [119, 120]. Two studies investigated glutamate-related processes in the frontal cortex of rats [115, 118]. Pascual et al. [118] showed reduced NR2B phosphorylation after two weeks of forced intermittent ethanol exposure in adolescents only. Using a 2-week ethanol vapor paradigm, Pian et al. [115] found different patterns of NMDAR subunit expression. These patterns were highly dependent on abstinence duration (0 h, 24 h, 2w), however, they only statistically compared results within rather than between age groups. Ethanol exposure was associated with decreased NR1 receptor expression in both age groups, but only the adult group showed a decrease in NR2A and NR2B expression. The NR1 and NR2A expression returned to normal during withdrawal, but in adults NR2B expression increased after two weeks of abstinence.

Conrad and Winder [109] assessed long-term potentiation (LTP) in the bed nucleus stria terminalis (BNST), a major output pathway of the amygdala towards the hypothalamus and thalamus. Voluntary ethanol exposure resulted in blunted LTP responses in the dorsolateral BNST regardless of age. However, all mice were socially isolated during the experiments to induce anxiety, so it is unclear whether the effects were solely due to ethanol exposure.

Two studies looked at glutamate receptor subunit expression in the hippocampus [108, 115]. Pian et al. [115] observed increased expression of NR1, NR2A, and NR2B in adults after 2 weeks of ethanol exposure. In adolescents, a reduction in NR2A expression was observed. After abstinence, adult levels returned to normal, while in adolescents, decreased NR1 and NR2A expression was seen after 24 h but an increased expression of these subunits was seen after 2 weeks of abstinence. These findings support regional specific effects of age group, with potentially increased sensitivity to the impact of alcohol on glutamatergic mediated hippocampal functioning in adolescents. Unlike expected, van Skike et al. [108] did not find effects of chronic intermittent ethanol exposure or withdrawal on NMDA receptor subunit expression in the hippocampus and cortex as a whole in adolescent and adult rats. The authors speculate that these null results might be associated with the exposure design (limited exposure and route of administration) and lack of withdrawal periods compared to Pian et al. [115].

In sum, there is limited and inconsistent evidence for age-related differences in glutamate function across seven studies. The direction of the observed age-related differences varies across regions, with evidence of both increased and decreased sensitivity to ethanol effects in adolescents compared to adults in the four studies that observed age-related differences.

##### GABA

GABA is the brain’s main inhibitory neurotransmitter. GABA_A_ receptors are a primary mediator of alcohol’s pharmacological effects [121]. A total of four studies looked at GABAergic functioning [108, 116, 122, 123]. Three of the four studies observed age-related differences.

One study investigated GABA-related processes in the (basolateral) amygdala, showing reduced GABA_A_ α1 and GAD67 (enzyme that converts Glutamate to GABA) mRNA expression in adult rats only, 60 days after 18-days ethanol exposure [116].

Two studies looked at the rat cortex as a whole [108, 122]. Van Skike et al. did not find effects of chronic intermittent ethanol exposure on GABA_A_ receptor expression [108]. Grobin et al. [122] showed that, while basal GABA_A_ receptor functioning was not affected by 1 month of chronic intermittent ethanol exposure, GABA_A_ receptors were less sensitive to the neurosteroid THDOC in adolescents. This neuromodulatory effect was not found in adults and did not persist after 33 days of abstinence. However, these results indicate that neurosteroids may play an indirect role in age differences in the GABAA receptor’s response to alcohol.

Two studies focused on the rat hippocampus [108, 124]. Fleming et al. [124] found age-specific effects of chronic intermittent ethanol exposure on hippocampal (dentate gyrus) GABA_A_ receptor functioning. Adolescent rats showed decreased tonic inhibitory current amplitudes after ethanol exposure, which was not the case for young adult and adult rats. Also, only the adolescents showed greater sensitivity to (ex vivo) acute ethanol exposure induced enhanced GABAergic tonic currents. The specificity of these effects to adolescent exposure might indicate adolescent vulnerability to ethanol-induced effects on the hippocampus; however, Van Skike et al. [108] did not find any effects of chronic intermittent ethanol exposure on GABA_A_ receptor expression in the hippocampus.

In sum, given the limited number of studies and lack of replicated effects, no clear conclusions can be drawn about the role of age on the effects of alcohol on GABAergic neurotransmission. Age-specific effects appear to be regionally distinct. The only available study found support for heightened adult sensitivity to ethanol in the amygdala. In contrast, one study found support for greater adolescent sensitivity in the hippocampus and whole cortex, whereas the other found no age-related differences.

##### Dopamine

The mesocorticolimbic dopamine system, with dopaminergic neurons in the ventral tegmental area (VTA) projecting to the NAc and prefrontal cortex, plays a key role in AUD, particularly through reward and motivational processes [14]. Only two studies investigated dopaminergic processes, focusing on the frontal cortex, NAc, and broader striatum [118, 125]. Both studies observed age-related differences in certain dopamine outcomes.

Carrara-Nascimento et al. [125] investigated acute effects of ethanol in adolescent and adult mice 5 days after a 15-day treatment with either ethanol or saline. In the PFC, ethanol pretreated adolescents showed reduced dopamine levels (DA) and related metabolites (DOPAC and HVA) in response to an acute ethanol challenge compared to ethanol pretreated adults and adolescent saline controls. In the NAc, there were no differences between pretreated adolescents and adults, but analyses within each age group revealed that ethanol-pretreatment with an acute challenge decreased DOPAC within the adolescent group. Results from the dorsal striatum also showed no differences between adolescents and adults. However, within the adolescent group, ethanol pre-treatment increased DOPAC and, within the adult group, it increased HVA. Pascual et al. [118] found similar results looking at the expression of DRD1 and DRD2 dopamine receptors after two weeks of chronic intermittent ethanol exposure in rats. In the NAc and dorsal striatum, DRD2 expression was reduced in adolescent compared to adult exposed rats, while both DRD1 and DRD2 expression were reduced in the frontal cortex.

These results suggest reduced alcohol-induced dopamine reactivity in adolescents in the PFC and NAc based on the two available studies, but more studies are warranted for a more detailed understanding of the relationship between age and dopamine receptor expression following chronic ethanol exposure.

##### Acetylcholine

Acetylcholine is a known neuromodulator of reward and cognition-related processes [126]. The composition and expression of nicotinic and muscarinic acetylcholine receptors have been implicated in various alcohol use-related behaviors [127, 128]. Only one study investigated cholinergic processes and observed age-related differences. Vetreno et al. [129] showed global reductions in choline acetyltransferase (ChAT; cholinergic cell marker) expression after adolescent onset, but not adult onset of forced intermittent binge-like exposure (20 days – every other day, 25 days abstinence).

#### Neuromodulatory processes

##### Neurodegeneration and neurodevelopment

Chronic alcohol consumption is thought to lead to brain damage by influencing processes involved in neurodegeneration and neurogenesis. The formation of addictive behaviors is paralleled by the formation of new axons and dendrites, strengthening specific neuronal pathways [130]. While brain morphology is commonly investigated in humans, it is a proxy of the impact of alcohol on the brain and therefore rarely studied in rodents. Five studies investigated facets of neurodegeneration or development in rodents [55, 65, 131–133]. All five studies observed age-related differences.

Huang et al. [131] showed reduced cerebral cortex mass in adolescent mice, but shortening of the corpus collosum in adults after 45 days of ethanol injections, suggesting some age-specific regional effects. Using an amino cupric silver staining, significant brain damage was revealed for both adolescent and adult rats after 4 days of binge-like ethanol exposure [132]. However, adolescents showed more damage in the olfactory-frontal cortex, perirhinal cortex, and piriform cortex.

Looking at hippocampal neurogenesis, ethanol exposure has been shown to initially reduce hippocampal neurogenesis in adult rodents, recovering after 1-month abstinence [134]. Compared to adults, neurogenesis in the dentate gyrus of the hippocampus was found to be reduced in adolescent exposed mice (Bromodeoxyuridine levels) [65] and rats (doublecortin levels) [133]. Lacaille et al. [65] also measured the expression level of genes involved in oxidative mechanisms after binge-like alcohol exposure. In whole brain samples, they found increased expression of genes involved in brain protection (i.e., gpx3, srxn1) in adults, but increased expression of genes involved in cell death (i.e., casp3) combined with decreased expression of genes involved in brain protection (i.e., gpx7, nudt15) in adolescents. Casp3 protein levels were also higher in the whole brain of adolescent exposed mice [65] and the adolescent dentate gyrus [133], suggesting more neurodegeneration and less neurogenesis in adolescents versus adults following ethanol consumption.

Cyclin-dependent kinase 5 (CDK5) is involved in axon, dendrite, and synapse formation and regulation. CDK5 is overexpressed in the prefrontal cortex and the NAc following exposure to substances of abuse including alcohol [135]. Moreover, CDK5 inhibition has been shown to reduce operant self-administration of alcohol in alcohol-dependent rats [136]. One study reported higher H4 acetylation of the CDK5 promoter in the PFC of adult versus adolescent ethanol-exposed rats during acute withdrawal, however, CDK5 mRNA expression was control-like after 2 weeks of abstinence [55].

In sum, strong conclusions cannot be drawn due to the limited number of studies and lack of replicated effects. However, preliminary evidence points to adolescent vulnerability to damage in the cortex, reduced neurogenesis, and increased neurodegeneration in the hippocampus and the cortex as a whole based on four of the five studies. In contrast, one study found support for adult vulnerability to ethanol’s effects axon, dendrite, and synapse formation and regulation.

##### Growth factors

Brain-derived neurotrophic factor (BNDF) and nerve growth factor (NGF) are involved in brain homeostasis and neural recovery [137, 138]. While ethanol exposure initially increases BDNF and NGF, chronic ethanol exposure seems to reduce BDNF and NGF levels and can thereby result in long-term brain damage and related cognitive problems [139, 140]. Four studies investigated growth factor expression in the frontal cortex [54, 55, 79, 80] and two studies also investigated the hippocampus [79, 80]. All four studies of the frontal cortex observed age-related differences. Neither study of the hippocampus observed age-related differences.

In rats, 30 weeks of chronic ethanol exposure reduced prefrontal mBDNF and β-NGF regardless of age, despite adolescents consuming more ethanol [80]. Moreover, the reduction of mBDNF was correlated with higher blood alcohol levels and was persistent up to 6–8 weeks abstinence. Interestingly, during acute withdrawal (48 h) adolescents but not adults temporarily showed control-like mBDNF levels. This might indicate an attempt to counteract neurodegeneration as a result of ethanol exposure in adolescents. These results were partially replicated using a shorter intermittent exposure paradigm (13 doses, 2 days on/off) [79]. While intoxication after chronic ethanol exposure reduced prefrontal BDNF, levels recovered after 3-weeks abstinence regardless of age. However, during acute withdrawal (24 h), BDNF was still reduced in early-adolescent onset rats, increased in adult-onset rats, but control-like in mid-adolescent onset-rats, suggesting slower recovery in younger animals. Looking at BDNF gene regulation, a similar study (8 doses, 2 days on/off) reported higher H3 demethylation but lower H4 acetylation of the BDNF promoter in the PFC of adult versus adolescent ethanol-exposed rats during acute withdrawal [55]. However, prefrontal BDNF mRNA expression returned to control levels after 2 weeks of abstinence. Interestingly, social housing may be protective, as reduced prefrontal BDNF was no longer observed in alcohol-exposed adolescent mice housed in environmentally enriched relative to standard conditions [54]. Two studies investigated hippocampal BDNF expression but reported no significant interactions between alcohol exposure and age group [79, 80].

In sum, the results of the four available studies suggest lower prefrontal BDNF during chronic alcohol use that recovers after abstinence regardless of age. However, the rate of recovery may be influenced by age with slower recovery in adolescents. In the two available studies, no age-related differences were observed in BDNF expression in the hippocampus.

##### Transcription factors

The transcription factors cFos and FosB are transiently upregulated in response to substance use, and ΔFosB accumulates after chronic exposure, particularly in striatal and other reward-related areas [141]. Two studies investigated cFos and FosB [55, 142] and one study ΔFosB related processes [111]. All three studies observed age-related differences.

After chronic ethanol exposure (8 doses, 2 days on/off), adolescent compared to adult rats showed increased prefrontal H3 and H4 acetylation of the cFos promotor region and increased H4 acetylation and H3 dimethylation of FosB promotor regions after acute abstinence [55]. Moreover, mRNA expression of FosB was elevated in adolescents but not adults after 2-weeks abstinence. The upregulating effects of an acute ethanol challenge on prefrontal cFos appears to reduce after chronic pre-treatment to a larger extent in adolescent than adult exposed mice [142]. This pattern of results was similar in the NAc, but desensitization to ethanol’s acute effects on cFos in the hippocampus was more pronounced in adults. Faria et al. [142] also looked at Egr-1 (transcription factor, indirect marker of neuronal activity and involved in neuroplasticity), showing a stronger reduction in Egr-1 expression in the PFC, NAc, and hippocampus of adolescent versus adults after repeated ethanol exposure. Regarding ∆FosB, Wille-Bille et al. [111] found increased ∆FosB in adolescent compared to adult rats in the prelimbic PFC, dorsomedial striatum, NAc core and shell, central amygdala nucleus capsular, and basolateral amygdala after 3 days per week 18 h ethanol exposure sessions for 6 weeks. In sum, the three available studies provide preliminary evidence for increased adolescent vulnerability to ethanol-induced long-term genetic (mRNA expression) and epigenetic (methylation) changes in mesocorticolimbic areas.

##### Immune factors

Ethanol is known to trigger immune responses in the brain (e.g., increase production of hemokines and cytokines), causing inflammation and oxidative stress [143–145]. Three studies examined immune factors [146–148]. Two of the three studies observed age-related differences.

Microglia remove damaged brain tissue and infectious agents and are key to the brain’s immune defense. Only one study investigated microglia levels [146]. Although direct comparisons between age groups were missing, both adolescent and adult rats showed less microglia in the hippocampus (CA and DG) and peri-entorhinal cortex, and more dysmorphic microglia in the hippocampus after 2 and 4 days of binge-like ethanol exposure [146]. Notably, age groups were matched on intoxication scores, with adolescents needing more ethanol to reach the same level of intoxication. An in silico transcriptome analysis of brain samples from mice after 4 days of 4 h/day drinking in the dark, suggest overexpression of neuroimmune pathways related to microglia action (toll-like receptor signaling, MAPK signaling, Jak-STAT signaling, T-cell signaling, and chemokine signaling) in adults that was not observed in adolescents, while adolescents consumed more ethanol [147]. Similarly, ethanol-exposed adult mice showed higher chemokine expression (CCL2/MCP-1) in the hippocampus, cerebral cortex, and cerebellum and higher cytokine expression (IL-6, but not TNF-α) in the cerebellum, while no chemokine or cytokine changes were observed in ethanol exposed adolescent mice [148]. Both adolescents and adults showed increased astrocyte levels in the hippocampus (CA1) and the cerebellum after ethanol exposure, but changes in astrocyte morphology were only observed in the adult hippocampus.

In sum, two of the studies found support for increased immune responses after ethanol exposure in adults compared to adolescents, whereas the one other study found no difference between the age groups.

##### HPA-axis functionality

Chronic stress and HPA-axis functionality have been associated with the maintenance of AUD (e.g., reinstatement drug seeking, withdrawal) [149]. Two studies investigated corticotropin-release factor (CRF) expression in rats [116, 150]. One study observed age-related differences and the other did not.

Falco et al. [116] found decreased CRF mRNA expression in the adult but not adolescent basolateral amygdala 2 months after 18-day restricted ethanol exposure. In contrast, Slawecki et al. did not find any interaction between age and treatment on CRF levels in the amygdala, as well as the frontal lobe, hippocampus, hypothalamus, and caudate 7 weeks after 10-days of ethanol vapor exposure.

No conclusions can be drawn. One study observed found support for reduced effects of ethanol on HPA-axis functionality compared to adults, whereas the other observed no difference between the age groups. Future studies using different (voluntary) exposure paradigms are needed to further investigate the effects of alcohol on HPA activity in relation to age of alcohol exposure.

##### Neuropeptides

Neuropeptides are a diverse class of proteins that have a modulatory function in many different processes, including but not limited to neurotransmission, stress, immune responses, homeostasis, and pain [151–153]. Only one study investigated neuropeptides in rats and observed age-related differences [150].

Slawecki et al. [150] specifically investigated neuropeptide-Y, substance-P, and interleukine expression in the frontal lobe, hippocampus, hypothalamus, dorsal striatum, and amygdala 7 weeks after 10-days of ethanol vapor exposure in rats [150]. Interactions between age and treatment were found for the hippocampus and caudate only. Ethanol-induced reductions in hippocampal neuropeptide-Y and increases in caudate neurokinine were more pronounced in adults compared to adolescents suggesting long-lasting effects of ethanol in adults but not adolescents.

##### Ethanol metabolism

The first metabolite of ethanol is acetaldehyde, which has been theorized to mediate the effects of ethanol on both brain and behavior [154]. Only one study investigated ethanol metabolism in the brain and did not observe age-related differences [155].

Rhoads et al. showed that despite the fact that adolescent rats consumed more alcohol brain catalase levels after 3-weeks of ethanol exposure (no abstinence) did not differ between adolescents and adults [155]. Although the general role of catalase in ethanol metabolism is small, catalase can oxidize ethanol to acetaldehyde in the brain, affecting elimination of ethanol after consumption [156, 157]. These findings may therefore imply that ethanol metabolism may not differ between adolescent and adult animals, which should be studied in a more direct manner.

##### Full proteome analysis

While the previously described studies focused on specific factors involved in neurotransmission, brain health, and plasticity, proteomics allows for the study of the full proteome in a specific region or tissue type. One study investigated the impact of age on ethanol-induced changes in the hippocampal proteome, observing age-related differences [158]. In this study, rats intermittently and voluntarily consumed beer for 1 month and the hippocampal proteome was analyzed after 2 weeks of abstinence. The results point to the involvement of many of the factors described above and imply age-specific effects of alcohol. Adult beer exposure increased citrate synthase (part of the citric acid, or Krebs, cycle) and fatty acid binding proteins (involved in membrane transport) compared to controls. Adolescent beer exposure increased cytoskeletal protein T-complex protein 1 subunit epsilon (TCP-1), involved in ATP-dependent protein folding, and reduced expression of a variety of other proteins involved in glycolysis, glutamate expression, aldehyde detoxification, protein degradation, and synaptogenesis, as well as neurotransmitter release. These more extensive changes suggest that the adolescent hippocampus might be more vulnerable to the effects of ethanol exposure, but more studies are needed to clarify and replicate these findings and extend the focus to different brain areas.

##### Neuronal activity and functioning

Ethanol-induced molecular changes may eventually change neuronal activity. Three studies investigated neuronal activity and functioning [89, 159, 160] using electrophysiological methods. All three studies observed age-related differences.

Galaj et al. [159] assessed firing patterns and the structure of pyramidal neurons in the L2 and L5 layers of the prelimbic cortex of the rat brain using ex vivo electrophysiological recordings and morphological staining. Following chronic intermittent ethanol exposure and brief abstinence (2 days), adolescents, but not adults, showed reduced amplitudes of spontaneous excitatory post-synaptic currents (sEPSCs) in L5 neurons compared to controls, indicating reductions in intrinsic excitability. In line with this, Dil staining showed increased thin spine ratios in the L5 layer in adolescents only. Age differences were more pronounced after prolonged abstinence (21 days), with adolescents showing reduced amplitude and frequency of sEPSCs in L5 neurons while adult’s L5 neurons showed augmented firing patterns (i.e., amplitude and frequency). Furthermore, adolescent rats showed decreased total spine density and non-thin spines, indicating less excitatory postsynaptic receptors in the L5 layer. In contrast, adults showed increases in spine density and non-thin spines.

Li et al. [160] examined the functioning of CA1 interneurons, which are important for learning and memory processes [161], in the rat hippocampus using ex vivo whole-cell recordings. After prolonged abstinence (20 days), voltage-gated A-type potassium channel (*I*_*A*_) conductance was measured. Differences emerged between age groups (although no statistical interaction effect was directly assessed): EtOH-exposed adolescents and adults both showed lower *I*_*A*_ mean peak amplitude compared to the respective control groups. However, adolescents also showed reduced *I*_*A*_ density and increased mean decay time, which decreased in adults. Furthermore, only adolescents showed increased depolarization required for activation compared to controls, which can result in higher interneuron firing rates in the CA1 region that could affect learning processes. Additional research is needed to connect these findings to behavioral measures of learning and memory.

Slawecki et al. [89] was the only study to use in vivo electroencephalogram (EEG) recordings with rats to examine function in the frontal and parietal cortex at different times during a 14-day vapor exposure period. During acute withdrawal (7–10 h abstinence period), following daily exposure no effects emerged in frontal cortical regions throughout the exposure period. In parietal regions, only adolescents showed increased high frequency (16–32 Hz and 32–50 Hz) power on days 8 and 12 compared to controls. Adolescent hyperexcitability during withdrawal may indicate increased arousal in adolescents compared to adults during withdrawal, but more studies linking brain activity to behavioral indices of withdrawal will allow for clearer interpretations.

Overall, strong conclusions cannot be drawn given the disparate paradigms and outcomes utilized. While adolescents and adults appear to differ in the effect of ethanol on neuronal firing, the meaning of these differences is not clear given the lack of connection between these findings and behavioral outcomes.

### Human studies

Four studies examined age-related differences of the effect of alcohol on brain or cognition in humans [162–165].

Müller-Oehring et al. [162] examined the moderating role of age on resting state functional connectivity and synchrony in the default mode, central executive, salience, emotion, and reward networks of the brain in a sample of no/low and heavier drinkers aged 12–21 years old. While the study did not compare discrete groups of adolescents and adults, analyses investigating the interaction between continuous age and alcohol exposure history were conducted which provide insight into the effect of alcohol use on functional brain networks from early adolescence to emerging adulthood. Regardless of age, no differences were observed between matched subgroups of no/low drinkers and moderate/heavy drinkers in the default mode, salience, or reward networks. However, in the central executive network, connectivity between the superior frontal gyrus (SFG) and insula increased with age in the no/low drinkers but not in heavier drinkers. Age-related strengthening of this fronto-limbic connection correlated with better performance on a delay discounting task in boys, suggesting that adolescent alcohol use may interfere with typical development of higher-level cognitive functions. In the emotion network, amygdala-medial parietal functional synchrony was reduced in the heavier drinkers compared to the no/low drinkers and exploratory analyses suggested that weaker amygdala-precuneus/posterior cingulate connectivity related to later stages of pubertal development in the no/low drinking group only. Interestingly, in the default mode (posterior cingulate-right hippocampus/amygdala) and emotional networks (amygdala, cerebellum), connectivity in regions that exhibited age-related desynchronization was negatively correlated with episodic memory performance in the heavy drinkers. These results give preliminary evidence that alcohol might have age-dependent effects on resting state connectivity and synchronization in the central executive, emotion, and default mode networks that could potentially interfere with normative maturation of these networks during adolescence.

Three studies examined age effects in alcohol-related implicit cognitions, specifically attentional bias [163, 165], alcohol approach bias [165], and implicit memory associations and explicit outcome expectancies [164]. Attentional bias refers to the preferential automatic allocation or maintenance of attention to alcohol-related cues compared to neutral cues which is correlated with alcohol use severity and craving [166]. McAteer et al. [163] measured attentional bias with eye tracking during presentation of alcohol and neutral stimuli in heavy and light drinkers in early adolescents (12–13 yrs), late adolescents (16–17 yrs), and young adults (18–21 yrs). Regardless of age, heavy drinkers spent longer fixating on alcohol cues compared to light drinkers. Cousijn et al. [165] measured attentional bias with an Alcohol Stroop task [167], comparing the speed of naming the print color of alcohol-related and control words. Consistent with the findings of McAteer et al. [163], adults and adolescents matched on monthly alcohol consumption showed similar levels of alcohol attentional bias. In the same study, Cousijn et al. [165] did not find any evidence for an approach bias towards alcohol cues in any age group.

Rooke and Hine [164] found evidence for age-related differences in implicit and explicit alcohol cognitions and their relationship with binge drinking. Using a teen-parent dyad design, adolescents (13–19 yrs) showed stronger memory associations in an associative phrase completion task and more positive explicit alcohol expectancies than adults. Interestingly, both explicit positive alcohol expectancies and implicit memory associations were a stronger predictor of binge drinking in adolescents compared to adults. It is important to note that adolescents also had higher levels of binge drinking than adults in the study.

Cousijn et al. [165] also investigated impulsivity, drinking motives, risky decision-making, interference control, and working memory. No age differences emerged in the cognitive functioning measures including risky decision-making (Columbia Card Task – “hot” version), interference control (Classical Stroop Task), or working memory (Self-Ordered Pointing Task). However, adolescents were more impulsive (Barrett Impulsiveness Scale) than adults and reported more enhancement motives. Importantly, impulsivity as well as social, coping, and enhancement motives of alcohol use correlated with alcohol use in both ages. However, age only moderated the relationship between social drinking motives and alcohol use-related problems (as measured by the Alcohol Use Disorder Identification Test), with a stronger positive association in adolescents compared to adults. Importantly, the adolescent group had a different pattern of drinking, with less drinking days per month but more drinks per episode than the adult group.

In summary, human evidence is largely missing, with no studies comparing more severe and dependent levels of alcohol use between adolescents and adults. The preliminary evidence is too weak and heterogeneous to draw conclusions, warranting future studies investigating the impact of age.

## Discussion

The current systematic review assessed the evidence for the moderating role of age in the effects of chronic alcohol use on the brain and cognition. The identified 59 rodent studies (Table 1) and 4 human studies (Table 2) provide initial evidence for the presence of age-related differences. Rodents exposed to ethanol during adolescence show both increased risk and resilience to the effects of ethanol depending on the outcome parameter. However, due to the high variability in the outcomes studied and the limited number of studies per outcome, conclusions should be considered preliminary. Moreover, brain and behavioral outcomes were mostly studied separately, with studies focusing on either brain or behavioral outcomes. The behavioral consequences of changes in certain brain outcomes still need to be investigated. Table 3 provides a comprehensive overview of the strength of the evidence for age-related differences for all outcomes. Below, we will discuss the most consistent patterns of results, make connections between the behavioral and neurobiological findings when possible, highlight strengths and limitations of the evidence base, and identify the most prominent research gaps.

**Table 3:** Overview of the strength of evidence for cognitive and neurobiological outcomes in animal studies.

Domain	# Studies	Strength of evidence for age-related differences
*Learning and memory*		
Conditioned taste aversion	4	*Limited* but *consistent* evidence for adolescents ↓
Conditioned place preference	4	*Inconsistent* evidence of age-related differences
Fear conditioning	5	Tone: *insufficient* evidence of age-related differences; Context: *limited* but *consistent* evidence adolescent ↑ impairment; Extinction: *limited* evidence of adults ↑ for context
Spatial learning and memory	6	*Inconsistent* evidence of age-related differences
Non-spatial learning and memory	6	MWM/SBM: *insufficient* evidence of age-related differences; Reward learning: *limited* evidence of no age-related difference in reward learning; NOR: *limited* evidence of adolescent ↑ impairment
*Executive function and higher-order cognition*		
Working memory	5	*Insufficient* evidence of age-related differences after chronic exposure; *Limited* but *consistent* evidence of chronic+acute challenge adolescent ↓
Attentional processes	2	*Limited* and *inconsistent* evidence for adolescent ↓
Cognitive flexibility	3	*Limited and inconsistent* evidence adolescent ↑ flexibility
Impulsivity	2	*Limited* and *inconsistent* evidence of age-related differences
Goal-directed behavior	2	*Limited* and *inconsistent* evidence of age-related differences
*Neurotransmission*		
Glutamate	7	*Limited* and *inconsistent* evidence of region-specific age-related differences
GABA	4	*Limited* and *inconsistent* evidence of age-related differences
Dopamine	2	NAc & PFC: *limited* but *consistent* evidence for adolescent **↓** dopamine reactivity; Striatum: *limited* and *inconsistent* evidence of age-related differences
Acetylcholine	1	*Limited* evidence for ↓ adolescent ChAT expression
*Neuromodulatory processes*		
Neurodegeneration and neurodevelopment	5	*Limited* evidence per outcome, but *consistent* ↑ adolescent sensitivity to neurodegeneration and impaired neurogenesis
Growth factors	4	FC: *Limited* but *consistent* age differences in BDNF during acute withdrawal; HC: *Limited* but *consistent* evidence for no age-related differences
Transcription factors	3	*Limited* but *consistent* evidence of adolescents ↑ epigenetic changes in FC and reward-related regions
Immune factors	3	*Limited* but *consistent* evidence of adults ↑ immune response
HPA-axis functionality	2	*Limited* and *inconsistent* evidence of age-related differences in amygdala; *Limited* evidence of no age-related differences in FC, HC, HYP and caudate
Neuropeptides	1	*Limited* evidence of adults ↑ reduction in HC and caudate
Ethanol metabolism in brain	1	*Limited* evidence of no age-related differences in brain catalase levels
Full proteome analysis	1	*Limited* evidence of adolescent ↑ changes
Neuronal activity and functioning	3	*Limited* but *consistent* evidence of age-related differences, direction of difference *inconsistent* depending on outcome measure
*Other behavioral outcomes*		
Social behavior	2	*Limited* and *inconsistent* evidence of age-related differences
Non-social anxiety	11	*Inconsistent* evidence of adolescent ↓

*Limited* = not enough studies; *Limited but consistent* = not enough studies but consistent direction of results; *Limited and inconsistent* = not enough studies and inconsistent directions of results; *Sufficient/Insufficient* = enough studies and results point in same direction; *Inconsistent* = enough studies but results point in different directions.

*BDNF* brain-derived neurotropic factor, *ChAT* choline Acetyltransferase, *FC* frontal cortex, *HC* hippocampus, *HYP* hypothalamus, *MWM* Morris water maze, *NAc* nucleus accumbens*, NOR* novel object recognition, *PFC* prefrontal cortex, *SBM* sand box maze.

### Patterns of results

Age-related differences in learning and memory-related processes appear to be highly domain specific. There is limited but fairly consistent evidence for adolescent-specific impairments in contextual fear conditioning, which could be related to hippocampal dysfunction. Results for other hippocampus-related memory processes such as spatial memory are mixed and largely based on forced exposure with acute challenge studies rather than voluntary long-term exposure to alcohol. The evidence base is currently insufficient to draw conclusions about the role of age in alcohol’s effects on non-spatial types of learning and memory. Alcohol generally did not impact performance in the non-spatial variants of the MWM and SBM paradigms or in reward-learning, but the results of the limited studies in the object-learning domain highlight potential impairments and the importance of age therein. For example, adolescents but not adults demonstrated impaired object memory in the only study using the novel object recognition task [65]. Acute challenges after chronic pre-exposure to alcohol also appear to impair performance in the working memory domain, with one study suggesting heightened adolescent sensitivity to working memory impairment [83]. Thus, although the domain-specific evidence is limited by the relative lack of research, overall patterns suggest that learning and memory functions that are primarily hippocampus-dependent may be differentially affected by adolescent compared to adult alcohol use. Studies focusing on neural hippocampal processes corroborate these findings, reporting more extensive changes in protein expression [158], less desensitization of cFos upregulation [142], larger changes in GABAa receptor subunit expression [124], longer lasting changes in NMDA receptor expression [115], and larger reductions in neurogenesis [65, 133] in the hippocampus of adolescent compared to adult ethanol-exposed rodents. On the other hand, ethanol-induced changes in the hippocampus recovered more quickly in younger animals after abstinence [150] and adolescent mice showed less signs of ethanol-induced neuroinflammation compared to adults [148].

Higher rates of adolescent alcohol use, especially binge drinking, may be facilitated by a heightened sensitivity to the rewarding properties of alcohol in combination with a reduced sensitivity to the negative effects of high doses [47]. In line with this, there is limited but consistent evidence that adolescents show less CTA in response to chronic ethanol and consequently voluntarily consume more ethanol [50]. Importantly, distinct vulnerability periods within adolescence for altered CTA may exist [168, 169], with early adolescents potentially being least sensitive to aversive effects. Future studies using chronic exposure paradigms comparing different stages of adolescence to adults are needed. In contrast to CTA, there is insufficient evidence of age-related differences in the motivational value of alcohol based on CPP paradigms, with only one of five studies reporting stronger CPP in adolescents than adults [52]. Adolescents may be more sensitive to the effects of environmental factors on the motivational value of alcohol than adults, as adolescents housed in enriched environments acquired CPP while those in standard housing did not, an effect that was not found in adults [54]. Evidence for environmentally enriched housing being protective against these changes in adolescents provides an important indication that environmental factors matter and are important factors to consider in future research on the motivational value of ethanol on both the behavioral and neural level. Complementary studies on the functioning of brain regions within the mesolimbic dopamine pathway and PFC, which play an important role in motivated behavior, indicate limited but consistent evidence for age-related differences. Adolescents showed less dopamine reactivity in the PFC and NAc compared to adults after chronic ethanol exposure. Furthermore, there is limited but consistent evidence that adolescents are more vulnerable to epigenetic changes in the frontal cortex and reward-related areas after chronic ethanol exposure. For instance, adolescents may be more sensitive to histone acetylation of transcription factors in motivational circuits underlying the rewarding effects of alcohol [55], which may contribute to addictive behaviors [170, 171]. Chronic alcohol use is also associated with lower BDNF levels in the PFC and subsequent increases in alcohol consumption, implicating BDNF as an important regulator of alcohol intake [172]. While evidence is limited, chronic alcohol use consistently reduced prefrontal BDNF in both age groups. However, the rate of recovery of BDNF levels after abstinence appears to be slower in adolescents.

Regarding executive functioning, there is limited but fairly consistent evidence from animal studies that adolescents are more vulnerable to long-term effects of chronic exposure on decision-making and are more impulsive than adults during acute intoxication and after prolonged abstinence following chronic exposure. Impulsivity is associated with functional alterations of the limbic cortico-striatal systems [91], with involvement of both the dopaminergic and serotonergic neurotransmitter systems [173]. While no studies investigating serotonergic activity were identified, the consistent reduction in dopamine reactivity observed in the PFC and NAc in adolescents compared to adults parallel the behavioral findings. There is also limited but fairly consistent evidence that adolescents are more resilient to impairments in cognitive flexibility than adults following chronic exposure to alcohol, and that adolescents may more easily regain control over their alcohol-seeking behavior than adults. These behavioral findings provide preliminary support for the paradox of adolescent risk and resilience in which adolescents are at once more at risk to develop harmful patterns of drinking, but are also more resilient in that they may be more equipped to flexibly change behavior and with time regain control over alcohol consumption. However, studies assessing processes that might be related to brain recovery provide little conclusive evidence for potential underlying mechanisms of these behavioral findings. While adolescents appear more vulnerable to ethanol-induced brain damage [131, 132], show reduced neurogenesis [65, 133], and show less changes in gene expression associated with brain recovery [65, 133], adults show relatively higher immune responses after repeated ethanol exposure [147, 148]. The limited evidence for adolescent resilience to alcohol’s effects on cognitive flexibility diverge from the conclusions of recent reviews that focused mostly on adolescent-specific research. Spear et al. [18] concluded that adolescents are more sensitive to impairments in cognitive flexibility; however, this was based on adolescent-only animal studies. Similarly, the systematic review of Carbia et al. [19] on the neuropsychological effects of binge drinking in adolescents and young adults also revealed impairments in executive functions, particularly inhibitory control. However, as pointed out by the authors, the lack of consideration of confounding variables (e.g., other drug use, psychiatric comorbidities, etc.) in the individual studies and the lack of prospective longitudinal studies limit our ability to causally interpret these results. This further highlights the difficulty of conducting human studies which elucidate causal associations of the effects of alcohol, and the need for animal research that directly compares adolescents to adults to bolster interpretation of findings from human research.

Only a few studies have investigated age-related differences in cognitive functioning in humans. These studies focused on mostly non-dependent users and studied different outcomes, including cognitive biases and implicit and explicit alcohol-related cognitions. Overall, there was limited but consistent evidence that age does not affect alcohol attentional or approach biases, with heavy drinkers in both age groups allocating more attention to alcohol cues compared to controls [163, 165]. In contrast, in line with a recent meta-analysis of the neurocognitive profile of binge-drinkers aged 10–24 [23], there is limited evidence that age affects alcohol associations. One study found age effects on implicit (memory associations) and explicit (expectancies) cognition in relation to alcohol use. Adolescents showed stronger memory associations and more positive expectancies than adults [164]. These expectancies were also predictive of higher binge drinking in adolescents but not adults, highlighting the importance of future research into age differences in alcohol-related cognitions and their consequences on alcohol consumption. However, the quality of the evidence was rated as weak based on the methodological design of the included studies.

Regarding anxiety-related outcomes, results are inconsistent across studies and paradigms. When age-differences are observed, adolescents often show reduced anxiety compared to adults during both acute withdrawal and sustained abstinence following chronic ethanol exposure. However, the direction of age-related effects of alcohol may also be anxiety-domain specific. In social settings, adults show reduced anxiety compared to adolescents. Research on the neurocircuitry of anxiety processes implicates the extended amygdala, especially the BNST, in anxiety behaviors with an emphasis on the role of GABAergic projections to the limbic, hindbrain, and cortical structures in rodents [174]. Despite adolescents showing less non-social anxiety than adults after ethanol exposure, no age-differences were observed for LTP in the BNST [109]. Also, GABA receptor expression in the hippocampus and whole cortex was not altered by ethanol exposure in either age group [108]. However, the anxiolytic effects of NMDA antagonists [175] also highlight the importance of glutamatergic activity in anxiety processes [176]. In line with behavioral findings, adolescents were less sensitive to changes in glutamate expression: adults showed heightened expression in the NAc, which has been suggested to underlie the higher levels of anxiety observed in adults compared to adolescents [106]. Importantly, across the various studies, different paradigms were used to assess anxiety, potentially contributing to the inconsistent results. Furthermore, most of the identified studies used a forced ethanol exposure paradigm. As alcohol-induced anxiety is likely also dependent on individual trait anxiety, voluntary consumption studies in high and low trait anxiety animals are important to further our understanding of the interaction between alcohol use and anxiety. Of note, the observed pattern suggestive of reduced anxiety in adolescents compared to adults diverges from conclusions of previous reviews such as Spear et al. [18] which concluded that adolescents are more likely to show augmented anxiety after alcohol exposure based on animal studies with adolescent animals only. Importantly, anxiety was included as a secondary outcome in this review because of the high comorbidity between anxiety disorders and alcohol addiction, warranting the inclusion of age-related differences in the relation between alcohol and anxiety. However, the search strategy was not specifically tailored to capturing all studies assessing age-related differences in the effect of alcohol on anxiety.

### Translational considerations, limitations, and future directions

The reviewed studies revealed a high degree of variability in study designs and outcomes, hindering integration and evaluation of research findings. We were unable to differentiate our conclusions based on drinking patterns (i.e., comparing binge drinking, heavy prolonged use, AUD). The prevalence of binge-drinking in adolescence is very high and is associated with neurocognitive alterations [177]. Studies investigating the potential differential impact of binge-drinking compared to non-binge-like heavy alcohol use in adolescence and adulthood are critical for understanding the risks of chronic binge-like exposure in adolescence, even if it does not progress to AUD.

It is also important to acknowledge the limitations of the choice of adolescent and adult age ranges in our inclusion criteria. Rodent studies had to include an adolescent group exposed to alcohol between the ages of PND 25–42 and an adult group exposed after age PND 65. Ontogenetic changes may still be occurring between PND 42–55, and this period may more closely correspond to late adolescence and emerging adulthood in humans (e.g., 18–25 years). Studies that compared animals in this post-pubertal but pre-adulthood age range were not reviewed. Studies investigating age-related differences in the effects of ethanol on brain and cognitive outcomes in emerging adulthood are also translationally valuable given the high rates and risky patterns of drinking observed during this developmental period [178]. Indeed, an important future direction is to examine whether there are distinct vulnerability periods within adolescence itself for the effects of ethanol on brain and cognitive outcomes. Given that emerging adulthood is a period of continued neurocognitive maturation and heightened neural plasticity, studies comparing this age range to older adults (e.g., over 30) are also necessary for a more thorough understanding of periods of risk and resilience to the effects of alcohol.

Furthermore, we did not conduct a risk of bias assessment to examine the methodological quality of the animal studies. The applicability and validity of the risk of bias tools for general animal intervention studies, such as the SYRCLE risk of bias tool [179], remain in question at the moment. The lack of standardized reporting in the literature for many of the criteria (e.g., process of randomizing animals into intervention groups) would lead to many studies being labeled with an ‘unclear risk of bias’. Furthermore, there is still a lack of empirical evidence regarding the impact of the criteria in these tools on bias [179, 180]. This is a significant limitation in evaluating the strength of the evidence for age-related differences based on the animal studies, which highlights the importance of more rigorous reporting standards in animal studies.

Moreover, most work is done in male rodents and is based on forced ethanol exposure regimes. In a recent opinion article, Field and Kersbergen [181] question the usefulness of these types of animal models to further our understanding of human substance use disorders (SUD). They argue that animal research has failed to deliver effective SUD treatment and that social, cultural, and other environmental factors crucial to human SUD are difficult, if not impossible, to model in animals. While it is clear that more sophisticated multi-symptom models incorporating social factors are needed to further our understanding of SUD and AUD specifically, a translational approach is still crucial in the context of investigating the more fundamental impact of alcohol use on brain and cognition. In humans, comparing the impact of alcohol use on brain and cognition between adolescents and adults is complicated by associations between age and cumulative exposure to alcohol; i.e., the older the individual, the longer and higher the overall exposure to alcohol. Although animal models may be limited in their ability to model every symptom of AUD, they can still provide critical insights into causal mechanisms underlying AUD by allowing direct control over alcohol exposure and in-depth investigation of brain mechanisms.

The intermittent voluntary access protocol resembles the patterns of alcohol use observed in humans, and also result in physiologically relevant levels of alcohol intake [182–184]. Only a minority of the studies included in this review employed a voluntary access protocol, with one study using beer instead of ethanol in water [158], which better accounts for the involvement of additional factors (e.g., sugar, taste) in the appeal of human alcohol consumption. Voluntary access protocols can also model behavioral aspects of addictive behavior such as loss of control over substance use and relapse [185–187], an important area in which little is known about the role of age. Ideally, one would also investigate choices between ethanol and alternative reinforcers, such as food or social interaction, that better mimic human decision-making processes [188]. However, studies on the effects of ethanol on social behavior are limited and show inconsistent results and studies assessing reward processes often lack a social reward component as an alternative reinforcer.

On a practical level, rodents mature quickly and choice-based exposure paradigms are more complex and time-consuming than most forced exposure paradigms. Consequently, by the time final behavioral measurements are recorded, both the adolescent and adult exposure groups have reached adulthood. To combat this, many of the included studies use forced ethanol exposure, such as ethanol vapor, to quickly expose rodents to very high doses of ethanol. Although the means and degrees of alcohol exposure may not directly translate to human patterns of alcohol use, such studies do allow for the assessment of the impact of high cumulative doses of ethanol within a relatively short period of time which allows for more time in the developmental window to test age-related differences in the outcomes. When considering the translational value of a study, it is therefore important to evaluate studies based on the goal, while not ignoring the practical constraints.

While human research is challenging due to the lack of experimental control and the inherent confounds in observational studies between age and alcohol exposure history, large-scale prospective longitudinal studies offer a gateway towards a better understanding. Comparisons of different trajectories of drinking from adolescence to adulthood (i.e., heavy drinking to light drinking, light drinking to heavy drinking, continuously heavy drinking, and continuously light drinking) could offer insight into the associated effects on cognitive and brain-related outcomes. Of course, different drinking trajectories are likely confounded with potentially relevant covariates which limits causal inference. Direct comparisons of low and heavy adolescent and adult drinkers, supported by a parallel animal model can help to bolster the causality of observed age-related differences in human studies. In addition, changes in legislation around the minimum age for alcohol consumption in some countries provide a unique opportunity to investigate how delaying alcohol use to later in adolescence or even young adulthood impacts cognitive functioning over time. Importantly, future studies investigating the moderating role of age in humans should carefully consider the impact of psychiatric comorbidities. While adolescence into young adulthood is the period in which mental health issues often emerge [189, 190], there is some evidence that the prevalence of comorbidities is higher in adults with AUD [95]. This is an important to control for when considering age-related differences on cognition and the brain given the evidence of altered cognitive functioning in other common mental illnesses [191, 192].

### Concluding remarks

The aim of this systematic review was to extend our understanding of adolescent risk and resilience to the effects of alcohol on brain and cognitive outcomes compared to adults. In comparison to recent existing reviews on the impact of alcohol on the adolescent brain and cognition [17–19, 22, 23], a strength of the current review is the direct comparison of the effects of chronic alcohol exposure during adolescence versus adulthood. This approach allows us to uncover *both* similarities and differences in the processes underlying alcohol use and dependence between adolescents and adults. However, due to the large degree of heterogeneity in the studies included in sample, designs, and outcomes, we were unable to perform meta-analytic synthesis techniques.

In conclusion, while the identified studies used varying paradigms and outcomes, key patterns of results emerged indicating a complex role of age, with evidence pointing towards both adolescent vulnerability and resilience. The evidence suggests adolescents may be more vulnerable than adults in domains that may promote heavy and binge drinking, including reduced sensitivity to aversive effects of high alcohol dosages, reduced dopaminergic neurotransmission in the NAc and PFC, greater neurodegeneration and impaired neurogenesis, and other neuromodulatory processes. At the same time, adolescents may be more resilient than adults to alcohol-induced impairments in domains which may promote recovery from heavy drinking, such as cognitive flexibility. However, in most domains, the evidence was too limited or inconsistent to draw clear conclusions. Importantly, human studies directly comparing adolescents and adults are largely missing. Recent reviews of longitudinal human research in adolescents, however, revealed consistent evidence of alterations to gray matter, and to a lesser extent white matter, structure in drinkers [17, 18], but also highlight the limited evidence available in the domains of neural and cognitive functioning in humans [17]. Future results from ongoing large-scale longitudinal neuroimaging studies like the ABCD study [193] will likely shed valuable light on the impact of alcohol use on the adolescent brain. However, our results also stress the need for direct comparisons with adult populations. Moreover, while the lack of experimental control and methodological constraints limit interpretations and causal attributions in human research, translational work aimed at connecting findings from animal models to humans is necessary to build upon the current knowledge base. Furthermore, the use of voluntary self-administration paradigms and incorporation of individual differences and environmental contexts are important steps forward in improving the validity of animal models of alcohol use and related problems. A more informed understanding of the effects of alcohol on adolescents compared to adults can further prevention efforts and better inform policy efforts aimed at minimizing harm during a crucial period for both social and cognitive development.

## Supplementary information

Appendix

## References

[1] Degenhardt L, Charlson F, Ferrari A, Santomauro D, Erskine H, Mantilla-Herrara A (2018). The global burden of disease attributable to alcohol and drug use in 195 countries and territories, 1990–2016: a systematic analysis for the Global Burden of Disease Study 2016. Lancet Psychiatry.

[2] Kohn R, Saxena S, Levav I, Saraceno B. The treatment gap in mental health care. World Health Organization; 2004. 10.1590/S0042-96862004001100011. PMC2623050 15640922

[3] Fleury MJ, Djouini A, Huỳnh C, Tremblay J, Ferland F, Ménard JM (2016). Remission from substance use disorders: a systematic review and meta-analysis. Drug Alcohol Depend.

[4] Hingson RW, Heeren T, Winter MR (2006). Age of alcohol-dependence onset: associations with severity of dependence and seeking treatment. Pediatrics.

[5] Hingson RW, Heeren T, Winter MR (2006). Age at drinking onset and alcohol dependence: age at onset, duration, and severity. Arch Pediatr Adolesc Med.

[6] American Psychiatric Association. Diagnostic and statistical manual of mental disorders. 5th edn. Arlington, VA: American Psychiatric Association; 2013. 10.1176/appi.books.9780890425596.dsm04.

[7] Conrod P, Nikolaou K (2016). Annual Research Review: On the developmental neuropsychology of substance use disorders. J Child Psychol Psychiatry Allied Discip.

[8] Spear LP (2015). Adolescent alcohol exposure: Are there separable vulnerable periods within adolescence?. Physiol Behav.

[9] Simon NW, Gregory TA, Wood J, Moghaddam B (2013). Differences in response initiation and behavioral flexibility between adolescent and adult rats. Behav Neurosci.

[10] Carroll LJ, Cassidy JD, Peloso PM, Borg J, von Holst H, Holm L, et al. Prognosis for mild traumatic brain injury: results of the WHO Collaborating Centre Task Force on Mild Traumatic Brain Injury. J Rehabil Med 2004(Suppl. 43):84–105. 10.1080/16501960410023859 15083873

[11] Mastwal S, Ye Y, Ren M, Jimenez DV, Martinowich K, Gerfen CR (2014). Phasic dopamine neuron activity elicits unique mesofrontal plasticity in adolescence. J Neurosci.

[12] Crone EA, Dahl RE (2012). Understanding adolescence as a period of social-affective engagement and goal flexibility. Nat Rev Neurosci.

[13] Vergés A, Haeny AM, Jackson KM, Bucholz KK, Grant JD, Trull TJ (2013). Refining the notion of maturing out: results from the national epidemiologic survey on alcohol and related conditions. Am J Public Health.

[14] Koob GF, Volkow ND (2010). Neurocircuitry of addiction. Neuropsychopharmacology.

[15] Seeley WW, Menon V, Schatzberg AF, Keller J, Glover GH, Kenna H (2007). Dissociable intrinsic connectivity networks for salience processing and executive control. J Neurosci.

[16] Ochsner KN, Gross JJ (2005). The cognitive control of emotion. Trends Cogn Sci..

[17] de Goede J, van der Mark-Reeuwijk KG, Braun KP, le Cessie S, Durston S, Engels RCME, et al. Alcohol and brain development in adolescents and young adults: a systematic review of the literature and advisory report of the health council of the Netherlands. Adv Nutr. 2021. 10.1093/advances/nmaa170. 10.1093/advances/nmaa170 33530096

[18] Spear LP (2018). Effects of adolescent alcohol consumption on the brain and behaviour. Nat Rev Neurosci.

[19] Carbia C, López-Caneda E, Corral M, Cadaveira F (2018). A systematic review of neuropsychological studies involving young binge drinkers. Neurosci Biobehav Rev..

[20] Feldstein Ewing SW, Sakhardande A, Blakemore SJ (2014). The effect of alcohol consumption on the adolescent brain: a systematic review of MRI and fMRI studies of alcohol-using youth. NeuroImage Clin.

[21] Squeglia LM, Boissoneault J, Van Skike CE, Nixon SJ, Matthews DB (2014). Age-related effects of alcohol from adolescent, adult, and aged populations using human and animal models. Alcohol Clin Exp Res.

[22] Lees B, Meredith LR, Kirkland AE, Bryant BE, Squeglia LM (2020). Effect of alcohol use on the adolescent brain and behavior. Pharm Biochem Behav.

[23] Lees B, Mewton L, Stapinski LA, Squeglia LM, Rae CD, Teesson M (2019). Neurobiological and cognitive profile of young binge drinkers: a systematic review and meta-analysis. Neuropsychol Rev..

[24] Cservenka A, Brumback T (2017). The burden of binge and heavy drinking on the brain: effects on adolescent and young adult neural structure and function. Front Psychol.

[25] Welch KA, Carson A, Lawrie SM (2013). Brain structure in adolescents and young adults with alcohol problems: systematic review of imaging studies. Alcohol Alcohol.

[26] Maeda K-I, Satoshi O, Hiroko T. Physiology of reproduction. Academic Press; 2000.

[27] Spear LP (2000). The adolescent brain and age-related behavioral manifestations. Neurosci Biobehav Rev.

[28] Burke AR, Miczek KA (2014). Stress in adolescence and drugs of abuse in rodent models: role of dopamine, CRF, and HPA axis. Psychopharmacology.

[29] Doremus-Fitzwater TL, Spear LP (2016). Reward-centricity and attenuated aversions: an adolescent phenotype emerging from studies in laboratory animals. Neurosci Biobehav Rev.

[30] Rajendran P, Spear LP. The effects of ethanol on spatial and nonspatial memory in adolescent and adult rats studied using an appetitive paradigm. In: Annals of the New York Academy of Sciences. New York Academy of Sciences; 2004. p. 441–4. 10.1196/annals.1308.060 15251925

[31] Morales M, Schatz KC, Anderson RI, Spear LP, Varlinskaya EI (2014). Conditioned taste aversion to ethanol in a social context: impact of age and sex. Behav Brain Res.

[32] Dumontheil I (2016). Adolescent brain development. Curr Opin Behav Sci.

[33] Shillington AM, Woodruff SI, Clapp JD, Reed MB, Lemus H (2012). Self-reported age of onset and telescoping for cigarettes, alcohol, and marijuana: across eight years of the national longitudinal survey of youth. J Child Adolesc Subst Abus.

[34] Livingston MD, Xu X, Komro KA (2016). Predictors of recall error in self-report of age at alcohol use onset. J Stud Alcohol Drugs.

[35] De Wit H (2009). Impulsivity as a determinant and consequence of drug use: a review of underlying processes. Addict Biol.

[36] Robinson TE, Berridge KC (1993). The neural basis of drug craving: an incentive-sensitization theory of addiction. Brain Res Rev.

[37] Rodriguiz RM, Wetsel WC. Assessments of cognitive deficits in mutant mice. In: Levin ED, Buccafusco JJ, editors. Animal models of cognitive impairment. CRC Press; 2006. p. 223–82. 21204369

[38] Leung RK, Toumbourou JW, Hemphill SA (2014). The effect of peer influence and selection processes on adolescent alcohol use: a systematic review of longitudinal studies. Health Psychol Rev.

[39] Cousijn J, Luijten M, Feldstein Ewing SW (2018). Adolescent resilience to addiction: a social plasticity hypothesis. Lancet Child Adolesc Heal.

[40] Kushner MG, Abrams K, Borchardt C (2000). The relationship between anxiety disorders and alcohol use disorders: a review of major perspectives and findings. Clin Psychol Rev.

[41] Robinson TE, Berridge KC (2008). Review. The incentive sensitization theory of addiction: some current issues. Philos Trans R Soc Lond B Biol Sci.

[42] Vanderschuren LJMJ, Pierce RC (2010). Sensitization processes in drug addiction. Curr Top Behav Neurosci.

[43] Gorey C, Kuhns L, Smaragdi E, Kroon E, Cousijn J (2019). Age-related differences in the impact of cannabis use on the brain and cognition: a systematic review. Eur Arch Psychiatry Clin Neurosci.

[44] Schweizer TA, Vogel-Sprott M, Danckert J, Roy EA, Skakum A, Broderick CE (2006). Neuropsychological profile of acute alcohol intoxication during ascending and descending blood alcohol concentrations. Neuropsychopharmacology.

[45] Ambrose ML, Bowden SC, Whelan G (2001). Working memory impairments in alcohol-dependent participants without clinical amnesia. Alcohol Clin Exp Res.

[46] Stavro K, Pelletier J, Potvin S (2013). Widespread and sustained cognitive deficits in alcoholism: a meta-analysis. Addict Biol.

[47] Spear LP (2011). Adolescent neurobehavioral characteristics, alcohol sensitivities, and intake: setting the stage for alcohol use disorders?. Child Dev Perspect.

[48] Holstein SE, Spanos M, Hodge CW (2011). Adolescent C57BL/6J mice show elevated alcohol intake, but reduced taste aversion, as compared to adult mice: a potential behavioral mechanism for binge drinking. Alcohol Clin Exp Res.

[49] Moore EM, Forrest RD, Boehm SL (2013). Genotype modulates age-related alterations in sensitivity to the aversive effects of ethanol: an eight inbred strain analysis of conditioned taste aversion. Genes, Brain Behav.

[50] Schramm-Sapyta NL, DiFeliceantonio AG, Foscue E, Glowacz S, Haseeb N, Wang N (2010). Aversive effects of ethanol in adolescent versus adult rats: potential causes and implication for future drinking. Alcohol Clin Exp Res.

[51] Pautassi RM, Myers M, Spear LP, Molina JC, Spear NE (2011). Ethanol induces second-order aversive conditioning in adolescent and adult rats. Alcohol.

[52] Carrara-Nascimento PF, Olive MF, Camarini R (2014). Ethanol pre-exposure during adolescence or adulthood increases ethanol intake but ethanol-induced conditioned place preference is enhanced only when pre-exposure occurs in adolescence. Dev Psychobiol.

[53] Leichtweis KS, Carvalho M, Morais-Silva G, Marin MT, Amaral VCS (2020). Short and prolonged maternal separation impacts on ethanol-related behaviors in rats: sex and age differences. Stress.

[54] Pautassi RM, Suárez AB, Hoffmann LB, Rueda AV, Rae M, Marianno P (2017). Effects of environmental enrichment upon ethanol-induced conditioned place preference and pre-frontal BDNF levels in adolescent and adult mice. Sci Rep.

[55] Pascual M, Do Couto BR, Alfonso-Loeches S, Aguilar MA, Rodriguez-Arias M, Guerri C (2012). Changes in histone acetylation in the prefrontal cortex of ethanol-exposed adolescent rats are associated with ethanol-induced place conditioning. Neuropharmacology.

[56] Peters J, Kalivas PW, Quirk GJ (2009). Extinction circuits for fear and addiction overlap in prefrontal cortex. Learn Mem..

[57] Antoniadis EA, McDonald RJ (2000). Amygdala, hippocampus and discriminative fear conditioning to context. Behav Brain Res.

[58] Marschner A, Kalisch R, Vervliet B, Vansteenwegen D, Büchel C (2008). Dissociable roles for the hippocampus and the amygdala in human cued versus context fear conditioning. J Neurosci.

[59] Orsini CA, Maren S (2012). Neural and cellular mechanisms of fear and extinction memory formation. Neurosci Biobehav Rev.

[60] Quirk GJ, Garcia R, González-Lima F (2006). Prefrontal mechanisms in extinction of conditioned fear. Biol Psychiatry.

[61] Bergstrom HC, McDonald CG, Smith RF (2006). Alcohol exposure during adolescence impairs auditory fear conditioning in adult Long-Evans rats. Physiol Behav.

[62] Broadwater M, Spear LP (2013). Consequences of ethanol exposure on cued and contextual fear conditioning and extinction differ depending on timing of exposure during adolescence or adulthood. Behav Brain Res.

[63] Broadwater M, Spear LP (2014). Consequences of adolescent or adult ethanol exposure on tone and context fear retention: effects of an acute ethanol challenge during conditioning. Alcohol Clin Exp Res.

[64] Broadwater M, Spear LP (2014). Tone conditioning potentiates rather than overshadows context fear in adult animals following adolescent ethanol exposure. Dev Psychobiol.

[65] Lacaille H, Duterte-Boucher D, Liot D, Vaudry H, Naassila M, Vaudry D (2015). Comparison of the deleterious effects of binge drinking-like alcohol exposure in adolescent and adult mice. J Neurochem.

[66] Markwiese BJ, Acheson SK, Levin ED, Wilson WA, Swartzwelder HS. Differential effects of ethanol on memory in adolescent and adult rats. In: Chapple L, editors. Alcoholism: clinical and Experimental Research. Blackwell Publishing Ltd; 1998. p. 416–21. 9581648

[67] Acheson SK, Ross EL, Swartzwelder HS (2001). Age-independent and dose-response effects of ethanol on spatial memory in rats. Alcohol.

[68] Sircar R, Sircar D (2005). Adolescent rats exposed to repeated ethanol treatment show lingering behavioral impairments. Alcohol Clin Exp Res.

[69] Swartzwelder HS, Hogan A, Risher ML, Swartzwelder RA, Wilson WA, Acheson SK (2014). Effect of sub-chronic intermittent ethanol exposure on spatial learning and ethanol sensitivity in adolescent and adult rats. Alcohol.

[70] Matthews DB, Watson MR, James K, Kastner A, Schneider A, Mittleman G (2019). The impact of low to moderate chronic intermittent ethanol exposure on behavioral endpoints in aged, adult, and adolescent rats. Alcohol.

[71] Galaj E, Barrera E, Morris D, Ma YY, Ranaldi R (2020). Aberrations in incentive learning and responding to heroin in male rats after adolescent or adult chronic binge-like alcohol exposure. Alcohol Clin Exp Res.

[72] Diamond A. Executive functions. 2013;64:135–68. 10.1146/annurev-psych-113011-143750. 10.1146/annurev-psych-113011-143750 PMC4084861 23020641

[73] Funahashi S, Andreau JM (2013). Prefrontal cortex and neural mechanisms of executive function. J Physiol Paris.

[74] Schindler AG, Tsutsui KT, Clark JJ (2014). Chronic alcohol intake during adolescence, but not adulthood, promotes persistent deficits in risk-based decision making. Alcohol Clin Exp Res.

[75] Risher ML, Fleming RL, Boutros N, Semenova S, Wilson WA, Levin ED (2013). Long-term effects of chronic intermittent ethanol exposure in adolescent and adult rats: radial-arm maze performance and operant food reinforced responding. PLoS ONE.

[76] Pickens CL, Cook A, Gaeddert B (2020). Dose-dependent effects of alcohol injections on omission-contingency learning have an inverted-U pattern. Behav Brain Res.

[77] Pickens CL, Kallenberger P, Pajser A, Fisher H (2019). Voluntary alcohol access during adolescence/early adulthood, but not during adulthood, causes faster omission contingency learning. Behav Brain Res.

[78] Mejia-Toiber J, Boutros N, Markou A, Semenova S (2014). Impulsive choice and anxiety-like behavior in adult rats exposed to chronic intermittent ethanol during adolescence and adulthood. Behav Brain Res.

[79] Fernandez GM, Lew BJ, Vedder LC, Savage LM (2017). Chronic intermittent ethanol exposure leads to alterations in brain-derived neurotrophic factor within the frontal cortex and impaired behavioral flexibility in both adolescent and adult rats. Neuroscience.

[80] Fernandez GM, Stewart WN, Savage LM. Chronic drinking during adolescence predisposes the adult rat for continued heavy drinking: neurotrophin and behavioral adaptation after long-term, continuous ethanol exposure. PLoS ONE 2016;11. 10.1371/journal.pone.0149987. 10.1371/journal.pone.0149987 PMC4773001 26930631

[81] Labots M, Cousijn J, Jolink LA, Leon Kenemans J, Vanderschuren LJMJ, Lesscher HMB (2018). Age-related differences in alcohol intake and control over alcohol seeking in rats. Front Psychiatry.

[82] Slawecki CJ, Ehlers CL (2005). Enhanced prepulse inhibition following adolescent ethanol exposure in Sprague-Dawley rats. Alcohol Clin Exp Res.

[83] White AM, Ghia AJ, Levin ED, Scott Swartzwelder H (2000). Binge pattern ethanol exposure in adolescent and adult rats: differential impact on subsequent responsiveness to ethanol. Alcohol Clin Exp Res.

[84] Baddeley A (1992). Working memory. Science.

[85] Olton DS, Samuelson RJ (1976). Remembrance of places passed: spatial memory in rats. J Exp Psychol Anim Behav Process.

[86] Deacon RMJ, Rawlins JNP (2006). T-maze alternation in the rodent. Nat Protoc.

[87] Knudsen EI (2007). Fundamental components of attention. Annu Rev Neurosci..

[88] Koch M, Schnitzler HU (1997). The acoustic startle response in rats—circuits mediating evocation, inhibition and potentiation. Behav Brain Res.

[89] Slawecki CJ, Roth J, Gilder A (2006). Neurobehavioral profiles during the acute phase of ethanol withdrawal in adolescent and adult Sprague-Dawley rats. Behav Brain Res.

[90] Cunha PJ, Nicastri S, de Andrade AG, Bolla KI (2010). The frontal assessment battery (FAB) reveals neurocognitive dysfunction in substance-dependent individuals in distinct executive domains: abstract reasoning, motor programming, and cognitive flexibility. Addict Behav.

[91] Jupp B, Dalley JW (2014). Convergent pharmacological mechanisms in impulsivity and addiction: insights from rodent models. Br J Pharm.

[92] Dickinson A, Balleine B (1994). Motivational control of goal-directed action. Anim Learn Behav.

[93] Tomie A, Sharma N (2013). Pavlovian sign-tracking model of alcohol abuse. Curr Drug Abus Rev.

[94] Tomie A, Jeffers P, Zito B. Sign-tracking model of the addiction blind spot. In: Tomie JMA, editors. Sign tracking and drug addiction. Maize Books; 2018. p. 8–34.

[95] Castillo-Carniglia A, Keyes KM, Hasin DS, Cerdá M (2019). Psychiatric comorbidities in alcohol use disorder. Lancet Psychiatry.

[96] Wolitzky-Taylor K, Bobova L, Zinbarg RE, Mineka S, Craske MG (2012). Longitudinal investigation of the impact of anxiety and mood disorders in adolescence on subsequent substance use disorder onset and vice versa. Addict Behav.

[97] Park J, Moghaddam B (2017). Impact of anxiety on prefrontal cortex encoding of cognitive flexibility. Neuroscience.

[98] Vytal KE, Cornwell BR, Letkiewicz AM, Arkin NE, Grillon C (2013). The complex interaction between anxiety and cognition: Insight from spatial and verbal working memory. Front Hum Neurosci.

[99] Prut L, Belzung C (2003). The open field as a paradigm to measure the effects of drugs on anxiety-like behaviors: a review. Eur J Pharmacol..

[100] Pellow S, Chopin P, File SE, Briley M (1985). Validation of open: closed arm entries in an elevated plus-maze as a measure of anxiety in the rat. J Neurosci Methods.

[101] Elsey JWB, Kindt M. Startle reflex. In: Zeigler-Hill V, Shackelford TK, editors. Encyclopedia of personality and individual differences. Springer International Publishing; 2018. p. 1–5.

[102] Crawley J, Goodwin FK (1980). Preliminary report of a simple animal behavior model for the anxiolytic effects of benzodiazepines. Pharm Biochem Behav.

[103] File SE, Seth P (2003). A review of 25 years of the social interaction test. Eur J Pharm.

[104] Misslin R, Ropartz P (1981). Responses in mice to a novel object author. Behavior.

[105] Njung’e K, Handley SL (1991). Evaluation of marble-burying behavior as a model of anxiety. Pharm Biochem Behav.

[106] Lee KM, Coelho MA, McGregor HA, Solton NR, Cohen M, Szumlinski KK (2016). Adolescent mice are resilient to alcohol withdrawal-induced anxiety and changes in indices of glutamate function within the nucleus accumbens. Front Cell Neurosci.

[107] Agoglia AE, Holstein SE, Reid G, Hodge CW (2015). CaMKIIα-GluA1 activity underlies vulnerability to adolescent binge alcohol drinking. Alcohol Clin Exp Res.

[108] Van Skike CE, Diaz-Granados JL, Matthews DB (2015). Chronic intermittent ethanol exposure produces persistent anxiety in adolescent and adult rats. Alcohol Clin Exp Res.

[109] Conrad KL, Winder DG (2011). Altered anxiety-like behavior and long-term potentiation in the bed nucleus of the stria terminalis in adult mice exposed to chronic social isolation, unpredictable stress, and ethanol beginning in adolescence. Alcohol.

[110] Slawecki CJ, Roth J (2004). Comparison of the onset of hypoactivity and anxiety-like behavior during alcohol withdrawal adolescent and adult rats. Alcohol Clin Exp Res.

[111] Wille-Bille A, de Olmos S, Marengo L, Chiner F, Pautassi RM (2017). Long-term ethanol self-administration induces ΔFosB in male and female adolescent, but not in adult, Wistar rats. Prog Neuro-Psychopharmacol Biol Psychiatry.

[112] Varlinskaya EI, Spear LP (2007). Chronic tolerance to the social consequences of ethanol in adolescent and adult Sprague-Dawley rats. Neurotoxicol Teratol.

[113] Morales M, Varlinskaya EI, Spear LP (2011). Age differences in the expression of acute and chronic tolerance to ethanol in male and female rats. Alcohol Clin Exp Res.

[114] Neuhofer D, Kalivas P (2018). Metaplasticity at the addicted tetrapartite synapse: a common denominator of drug induced adaptations and potential treatment target for addiction. Neurobiol Learn Mem.

[115] Pian JP, Criado JR, Milner R, Ehlers CL (2010). N-methyl-d-aspartate receptor subunit expression in adult and adolescent brain following chronic ethanol exposure. Neuroscience.

[116] Falco AM, Bergstrom HC, Bachus SE, Smith RF (2009). Persisting changes in basolateral amygdala mRNAs after chronic ethanol consumption. Physiol Behav.

[117] Chin VS, Van Skike CE, Berry RB, Kirk RE, Diaz-Granados J, Matthews DB (2011). Effect of acute ethanol and acute allopregnanolone on spatial memory in adolescent and adult rats. Alcohol.

[118] Pascual M, Boix J, Felipo V, Guerri C (2009). Repeated alcohol administration during adolescence causes changes in the mesolimbic dopaminergic and glutamatergic systems and promotes alcohol intake in the adult rat. J Neurochem.

[119] Akkus F, Mihov Y, Treyer V, Ametamey SM, Johayem A, Senn S, et al. Metabotropic glutamate receptor 5 binding in male patients with alcohol use disorder. Transl Psychiatry 2018;8. 10.1038/s41398-017-0066-6. 10.1038/s41398-017-0066-6 PMC5802584 29317611

[120] Leurquin-Sterk G, Ceccarini J, Crunelle CL, De Laat B, Verbeek J, Deman S (2018). Lower limbic metabotropic glutamate receptor 5 availability in alcohol dependence. J Nucl Med.

[121] Davies M (2003). The role of GABAA receptors in mediating the effects of alcohol in the central nervous system. J Psychiatry Neurosci.

[122] Grobin AC, Matthews DB, Montoya D, Wilson WA, Morrow AL, Swartzwelder HS (2001). Age-related differences in neurosteroid potentiation of muscimol-stimulated 36Cl- flux following chronic ethanol treatment. Neuroscience.

[123] Fleming RL, Acheson SK, Moore SD, Wilson WA, Swartzwelder HS (2011). GABA transport modulates the ethanol sensitivity of tonic inhibition in the rat dentate gyrus. Alcohol.

[124] Fleming RL, Li Q, Risher ML, Sexton HG, Moore SD, Wilson WA (2013). Binge-pattern ethanol exposure during adolescence, but not adulthood, causes persistent changes in GABAA receptor-mediated tonic inhibition in dentate granule cells. Alcohol Clin Exp Res.

[125] Carrara-Nascimento PF, Hoffmann LB, Flório JC, Planeta CS, Camarini R (2020). Effects of ethanol exposure during adolescence or adulthood on locomotor sensitization and dopamine levels in the reward system. Front Behav Neurosci.

[126] Picciotto MR, Higley MJ, Mineur YS (2012). Acetylcholine as a neuromodulator: cholinergic signaling shapes nervous system function and behavior. Neuron.

[127] Wu J, Gao M, Taylor DH (2014). Neuronal nicotinic acetylcholine receptors are important targets for alcohol reward and dependence. Acta Pharmacol Sin..

[128] Walker LC, Berizzi AE, Chen NA, Rueda P, Perreau VM, Huckstep K (2020). Acetylcholine muscarinic M4 receptors as a therapeutic target for alcohol use disorder: converging evidence from humans and rodents. Biol Psychiatry.

[129] Vetreno RP, Broadwater M, Liu W, Spear LP, Crews FT (2014). Adolescent, but not adult, binge ethanol exposure leads to persistent global reductions of choline acetyltransferase expressing neurons in brain. PLoS ONE.

[130] Koob GF, Volkow ND (2016). Neurobiology of addiction: a neurocircuitry analysis. Lancet Psychiatry.

[131] Huang C, Titus JA, Bell RL, Kapros T, Chen J, Huang R (2012). A mouse model for adolescent alcohol abuse: stunted growth and effects in brain. Alcohol Clin Exp Res.

[132] Crews FT, Braun CJ, Hoplight B, Switzer RC, Knapp DJ (2000). Binge ethanol consumption causes differential brain damage in young adolescent rats compared with adult rats. Alcohol Clin Exp Res.

[133] Broadwater MA, Liu W, Crews FT, Spear LP (2014). Persistent loss of hippocampal neurogenesis and increased cell death following adolescent, but not adult, chronic ethanol exposure. Dev Neurosci.

[134] Nixon K, Kim DH, Potts EN, He J, Crews FT (2008). Distinct cell proliferation events during abstinence after alcohol dependence: microglia proliferation precedes neurogenesis. Neurobiol Dis.

[135] Camp MC, Mayfield RD, McCracken M, McCracken L, Alcantara AA (2006). Neuroadaptations of Cdk5 in cholinergic interneurons of the nucleus accumbens and prefrontal cortex of inbred alcohol-preferring rats following voluntary alcohol drinking. Alcohol Clin Exp Res.

[136] Goulding SP, de Guglielmo G, Carrette LLG, George O, Contet C (2019). Systemic administration of the cyclin-dependent kinase inhibitor (S)-CR8 selectively reduces escalated ethanol intake in dependent rats. Alcohol Clin Exp Res.

[137] Joe KH, Kim YK, Kim TS, Roh SW, Choi SW, Kim YB (2007). Decreased plasma brain-derived neurotrophic factor levels in patients with alcohol dependence. Alcohol Clin Exp Res.

[138] Huang MC, Chen CH, Chen CH, Liu SC, Ho CJ, Shen WW (2008). Alterations of serum brain-derived neurotrophic factor levels in early alcohol withdrawal. Alcohol Alcohol.

[139] Miller R, King MA, Heaton MB, Walker DW (2002). The effects of chronic ethanol consumption on neurotrophins and their receptors in the rat hippocampus and basal forebrain. Brain Res.

[140] Vetreno RP, Crews FT (2015). Binge ethanol exposure during adolescence leads to a persistent loss of neurogenesis in the dorsal and ventral hippocampus that is associated with impaired adult cognitive functioning. Front Neurosci.

[141] Robison AJ, Nestler EJ (2011). Transcriptional and epigenetic mechanisms of addiction. Nat Rev Neurosci..

[142] Faria RR, Lima Rueda AV, Sayuri C, Soares SL, Malta MB, Carrara-Nascimento PF (2008). Environmental modulation of ethanol-induced locomotor activity: Correlation with neuronal activity in distinct brain regions of adolescent and adult Swiss mice. Brain Res.

[143] Crews FT, Bechara R, Brown LA, Guidot DM, Mandrekar P, Oak S, et al. Cytokines and alcohol. In: Alcoholism: clinical and experimental research. John Wiley & Sons, Ltd; 2006. p. 720–30. 10.1111/j.1530-0277.2006.00084.x 16573591

[144] Davis RL, Syapin PJ (2004). Chronic ethanol inhibits CXC chemokine ligand 10 production in human A172 astroglia and astroglial-mediated leukocyte chemotaxis. Neurosci Lett.

[145] Knapp DJ, Crews FT (1999). Induction of cyclooxygenase-2 in brain during acute and chronic ethanol treatment and ethanol withdrawal. Alcohol Clin Exp Res.

[146] Marshall SA, McClain JA, Wooden JI, Nixon K (2020). Microglia dystrophy following binge-like alcohol exposure in adolescent and adult male rats. Front Neuroanat.

[147] Agrawal RG, Owen JA, Levin PS, Hewetson A, Berman AE, Franklin SR (2014). Bioinformatics analyses reveal age-specific neuroimmune modulation as a target for treatment of high ethanol drinking. Alcohol Clin Exp Res.

[148] Kane CJM, Phelan KD, Douglas JC, Wagoner G, Johnson JW, Xu J (2014). Effects of ethanol on immune response in the brain: region-specific changes in adolescent versus adult mice. Alcohol Clin Exp Res.

[149] Blaine SK, Sinha R (2017). Alcohol, stress, and glucocorticoids: from risk to dependence and relapse in alcohol use disorders. Neuropharmacology.

[150] Slawecki CJ, Jiménez-Vasquez P, Mathé AA, Ehlers CL (2005). Effect of ethanol on brain neuropeptides in adolescent and adult rats. J Stud Alcohol.

[151] van den Pol AN (2012). Neuropeptide transmission in brain circuits. Neuron.

[152] Souza-Moreira L, Campos-Salinas J, Caro M, Gonzalez-Rey E (2011). Neuropeptides as pleiotropic modulators of the immune response. Neuroendocrinology.

[153] Carniglia L, Ramírez D, Durand D, Saba J, Turati J, Caruso C, et al. Neuropeptides and microglial activation in inflammation, pain, and neurodegenerative diseases. Mediators Inflamm. 2017;2017. 10.1155/2017/5048616. 10.1155/2017/5048616 PMC5244030 28154473

[154] Hipolito L, Sanchez M, Polache A, Granero L (2007). Brain metabolism of ethanol and alcoholism: an update. Curr Drug Metab.

[155] Rhoads DE, Contreras C, Fathalla S. Brain levels of catalase remain constant through strain, developmental, and chronic alcohol challenges. Enzyme Res. 2012;2012. 10.1155/2012/572939. 10.1155/2012/572939 PMC3420129 22919469

[156] Vasiliou V, Ziegler TL, Bludeau P, Petersen DR, Gonzalez FJ, Deitrich RA (2006). CYP2E1 and catalase influence ethanol sensitivity in the central nervous system. Pharmacogenet Genomics.

[157] Zimatkin SM, Buben AI (2007). Ethanol oxidation in the living brain. Alcohol Alcohol.

[158] Hargreaves GA, Quinn H, Kashem MA, Matsumoto I, McGregor IS (2009). Proteomic analysis demonstrates adolescent vulnerability to lasting hippocampal changes following chronic alcohol consumption. Alcohol Clin Exp Res.

[159] Galaj E, Guo C, Huang D, Ranaldi R, Ma YY (2020). Contrasting effects of adolescent and early-adult ethanol exposure on prelimbic cortical pyramidal neurons. Drug Alcohol Depend.

[160] Li Q, Fleming RL, Acheson SK, Madison RD, Moore SD, Risher ML (2013). Long-term modulation of A-type K+ conductances in hippocampal CA1 interneurons in rats after chronic intermittent ethanol exposure during adolescence or adulthood. Alcohol Clin Exp Res.

[161] Artinian J, Lacaille JC (2018). Disinhibition in learning and memory circuits: new vistas for somatostatin interneurons and long-term synaptic plasticity. Brain Res Bull.

[162] Müller-Oehring EM, Kwon D, Nagel BJ, Sullivan EV, Chu W, Rohlfing T (2018). Influences of age, sex, and moderate alcohol drinking on the intrinsic functional architecture of adolescent brains. Cereb Cortex.

[163] McAteer AM, Hanna D, Curran D (2018). Age-related differences in alcohol attention bias: a cross-sectional study. Psychopharmacology.

[164] Rooke SE, Hine DW (2011). A dual process account of adolescent and adult binge drinking. Addict Behav.

[165] Cousijn J, Green KH, Labots M, Vanderschuren LJMJ, Kenemans JL, Lesscher HMB (2020). Motivational and control mechanisms underlying adolescent versus adult alcohol use. NeuroSci.

[166] Field M, Cox WM (2008). Attentional bias in addictive behaviors: a review of its development, causes, and consequences. Drug Alcohol Depend.

[167] Cousijn J, van Benthem P, van der Schee E, Spijkerman R (2015). Motivational and control mechanisms underlying adolescent cannabis use disorders: a prospective study. Dev Cogn Neurosci.

[168] Saalfield J, Spear L (2015). Consequences of repeated ethanol exposure during early or late adolescence on conditioned taste aversions in rats. Dev Cogn Neurosci.

[169] Saalfield J, Spear L (2016). The ontogeny of ethanol aversion. Physiol Behav.

[170] McQuown SC, Wood MA (2010). Epigenetic regulation in substance use disorders. Curr Psychiatry Rep.

[171] Renthal W, Nestler EJ (2008). Epigenetic mechanisms in drug addiction. Trends Mol Med..

[172] Logrip ML, Barak S, Warnault V, Ron D (2015). Corticostriatal BDNF and alcohol addiction. Brain Res.

[173] Dalley JW, Roiser JP (2012). Dopamine, serotonin and impulsivity. Neuroscience.

[174] Robinson OJ, Pike AC, Cornwell B, Grillon C (2019). The translational neural circuitry of anxiety. J Neurol Neurosurg Psychiatry.

[175] Martínez G, Ropero C, Funes A, Flores E, Blotta C, Landa AI (2002). Effects of selective NMDA and non-NMDA blockade in the nucleus accumbens on the plus-maze test. Physiol Behav.

[176] Bergink V, Van Megen HJGM, Westenberg HGM (2004). Glutamate and anxiety. Eur Neuropsychopharmacol..

[177] Scott AJ, Jordan M, Lueras BJN (2018). Effects of binge drinking on the developing brain. Alcohol Res.

[178] Krieger H, Young CM, Anthenien AM, Neighbors C (2018). The epidemiology of binge drinking among college-age individuals in the United States. Alcohol Res.

[179] Hooijmans CR, Rovers MM, De Vries RBM, Leenaars M, Ritskes-Hoitinga M, Langendam MW (2014). SYRCLE’s risk of bias tool for animal studies. BMC Med Res Methodol.

[180] Krauth D, Woodruff TJ, Bero L (2013). Instruments for assessing risk of bias and other methodological criteria of published animal studies: a systematic review. Environ Health Perspect.

[181] Field M, Kersbergen I (2020). Are animal models of addiction useful?. Addiction.

[182] Simms JA, Steensland P, Medina B, Abernathy KE, Chandler LJ, Wise R (2008). Intermittent access to 20% ethanol induces high ethanol consumption in Long-Evans and Wistar rats. Alcohol Clin Exp Res.

[183] Rhodes JS, Best K, Belknap JK, Finn DA, Crabbe JC (2005). Evaluation of a simple model of ethanol drinking to intoxication in C57BL/6J mice. Physiol Behav.

[184] Spoelder M, Hesseling P, Baars AM, Lozeman-van’t Klooster JG, Rotte MD, Vanderschuren LJMJ (2015). Individual variation in alcohol intake predicts reinforcement, motivation, and compulsive alcohol use in rats. Alcohol Clin Exp Res.

[185] Fredriksson I, Venniro M, Reiner DJ, Chow JJ, Bossert JM, Shaham Y (2021). Animal models of drug relapse and craving after voluntary abstinence: a review. Pharm Rev.

[186] Vanderschuren LJMJ, Ahmed SH. Animal models of the behavioral symptoms of substance use disorders. Cold Spring Harb Perspect Med. 2021;11:a040287. 10.1101/cshperspect.a040287 PMC8327824 32513674

[187] Kuhn BN, Kalivas PW, Bobadilla AC. Understanding addiction using animal models. Front Behav Neurosci. 2019;13. 10.3389/fnbeh.2019.00262. 10.3389/fnbeh.2019.00262 PMC6895146 31849622

[188] Venniro M, Shaham Y (2020). An operant social self-administration and choice model in rats. Nat Protoc.

[189] Thapar A, Collishaw S, Pine DS, Thapar AK (2012). Depression in adolescence. Lancet.

[190] Costello EJ, Egger H, Angold A (2005). 10-Year research update review: the epidemiology of child and adolescent psychiatric disorders: I. Methods and public health burden. J Am Acad Child Adolesc Psychiatry.

[191] Snyder HR, Kaiser RH, Whisman MA, Turner AEJ, Guild RM, Munakata Y. Opposite effects of anxiety and depressive symptoms on executive function: the case of selecting among competing options. 2014;28:893–902. 10.1080/026999312013859568. 10.1080/02699931.2013.859568 PMC4020950 24295077

[192] McDermott LM, Ebmeier KP (2009). A meta-analysis of depression severity and cognitive function. J Affect Disord.

[193] Volkow ND, Koob GF, Croyle RT, Bianchi DW, Gordon JA, Koroshetz WJ (2018). The conception of the ABCD study: from substance use to a broad NIH collaboration. Dev Cogn Neurosci.

