# Supplementary material for: Age-related differences in the effect of chronic alcohol on cognition and the brain: a systematic review
Source: Transl Psychiatry. 2022 Aug 25;12:345. doi: 10.1038/s41398-022-02100-y (PMC9411553; doi:10.1038/s41398-022-02100-y)
Supplement: Supplementary file 1 — Appendix [file 41398_2022_2100_MOESM1_ESM.docx]

**Appendix**

Our literature search used Medline from the National Library of Medicine (United States of America), PsycINFO from the American Psychological Association, and Cochrane Library from John Wiley & Sons. The initial hits were downloaded from the databases (see Table 4, 5, & 6 for search strategy).

Medline (4,007), PsycINFO (2,738), and Cochrane Library (2,648) databases generated a total of blank hits, before deduplication. After the initial download, the results were uploaded to Zotero reference manager. Then, results were combined and de-duplicated using a filter that displayed the creator, year, issue, pages, and volume of each article and then, using the merge function if necessary. Following this, results were uploaded to Rayyan for blinded review. All articles were reviewed by at least two of the following assessors, LK, JC, CG, and GM. Consensus for discrepancies was reached by all assessors (CG, GM, LK, & JC). Duplicates that were missed in Zotero were resolved in Rayyan. The total number of references after deduplication was 7,229. Results from the review were downloaded to excel (see Fig 2. for entire screening process).

**Table 4** Search syntax from Medline database, last accessed on February 3, 2021

| **Medline**  *Ovid MEDLINE(R) and Epub Ahead of Print, In-Process & Other Non-Indexed Citations, and Daily 1946 to* February 1, 2021    **#1 Alcohol**  binge drinking/ OR alcoholic intoxication/ OR ethanol/ OR (EtOH OR ethanol OR alcohol).ti,ab,kf.    **#2 Cognition**  cognitive dysfunction/ OR cognition/ OR neuropsychology/ OR decision making/ OR memory/ OR memory, long-term/ OR memory, short-term/ OR spatial memory/ OR spatial learning/ OR neurocognition/ OR inhibition/ OR (cognit* OR brain* OR memory OR executive function* OR intellectual* function* OR learning OR conditioning OR aversion  OR processing speed OR inhibition OR locomotor OR craving OR neuropsychol* OR neurocognit*).ti,ab,kf.    **#3 Adults and adolescents**  adolescent/ OR young adult/ OR adult/ OR (adolesc* OR adult*).ti,ab,kf.    **#4 Study type**  longitudinal studies/ OR cross-sectional studies/ OR age factors/ OR (longitud* OR cross-sectional* OR group difference* OR vehicle OR age-related difference* OR age difference* OR (adolesc* ADJ3 adult*) OR follow up OR followup).ti,ab,kf.  **1 AND 2 AND 3 AND 4** |
| --- |

**Table 5** Search syntax from PsycINFO database, last accessed on February 3, 2021

| **PsycINFO**  ***Ovid, 1806 to*** January Week 4 2021    **#1 Alcohol**  alcohol drinking patterns/ OR binge drinking/ OR ethanol/ OR (EtOH OR ethanol OR alcohol).ti,ab,id.  **#2 Cognition**  cognitive impairment/ OR cognitive ability/ OR neuropsychology/ OR cognitive assessment/ OR decision making/ OR memory/ OR long term memory/ OR short term memory/ OR spatial memory/ OR neurocognition/ OR response inhibition/ OR behavioral inhibition/ OR prepulse inhibition/ OR proactive inhibition/ OR retroactive inhibition/ OR (cognit* OR brain* OR memory OR executive function* OR intellectual* function* OR learning OR conditioning OR aversion OR processing speed OR inhibition OR locomotor OR craving OR neuropsychol* OR neurocognit*).ti,ab,id.    **#3 Adults and adolescents**  (adolescence 13 17 yrs OR young adulthood 18 29 yrs OR adulthood OR thirties).ag. OR (adolesc* OR adult*).ti,ab,id.    **#4 Study type**  (longitudinal study OR followup study).md. OR longitudinal studies/ OR age differences/ OR group differences/ OR (longitud* OR cross-sectional* OR group difference* OR vehicle OR age-related difference* OR age difference* OR (adolesc* ADJ3 adult*) OR follow up OR followup).ti,ab,id.    **1 AND 2 AND 3 AND 4** |
| --- |

**Table 6** Search syntax from Cochrane Library database, last accessed on February 3, 2021

| **Cochrane Library** *Wiley Interscience*    **#1 Alcohol**  ("alcohol" OR “EtOH” OR "ethanol"):ti,ab,kw  **#2 Cognition**  ("cognit*" OR "brain*" OR "memory" OR "executive function*" OR "intellectual* function*" OR "learning" OR "conditioning" OR "aversion" OR "processing speed" OR “inhibition” OR  "locomotor" OR "craving" OR "neuropsychol*" OR "neurocognit*"):ti,ab,kw    **#3 Adults and adolescents**  ("adolesc*" OR "adult*"):ti,ab,kw  **#4 Study type**  ("longitud*" OR "cross-sectional*" OR "group difference*" OR "vehicle" OR "age-related difference*" OR "age difference*" OR ("adolesc*" NEAR/2 "adult*") OR "follow up" OR "followup"):ti,ab,kw    **1 AND 2 AND 3 AND 4** |
| --- |
